# Phenotypic Evaluation and Genetic Analysis of Seedling Emergence in a Global Collection of Wheat Genotypes (*Triticum aestivum* L.) Under Limited Water Availability

**DOI:** 10.3389/fpls.2021.796176

**Published:** 2021-12-24

**Authors:** Michael G. Francki, Grantley S. Stainer, Esther Walker, Gregory J. Rebetzke, Katia T. Stefanova, Robert J. French

**Affiliations:** ^1^Department of Primary Industries and Regional Development, South Perth, WA, Australia; ^2^State Agricultural Biotechnology Centre, Murdoch University, Murdoch, WA, Australia; ^3^Department of Primary Industries and Regional Development, Merredin, WA, Australia; ^4^Commonwealth Scientific and Industrial Research Organisation, Agriculture and Food, Canberra, ACT, Australia

**Keywords:** emergence, germination, dormancy, QTL, environment, genotype-by-environment interactions, genes

## Abstract

The challenge in establishing an early-sown wheat crop in southern Australia is the need for consistently high seedling emergence when sowing deep in subsoil moisture (>10 cm) or into dry top-soil (4 cm). However, the latter is strongly reliant on a minimum soil water availability to ensure successful seedling emergence. This study aimed to: (1) evaluate 233 Australian and selected international wheat genotypes for consistently high seedling emergence under limited soil water availability when sown in 4 cm of top-soil in field and glasshouse (GH) studies; (2) ascertain genetic loci associated with phenotypic variation using a genome-wide association study (GWAS); and (3) compare across loci for traits controlling coleoptile characteristics, germination, dormancy, and pre-harvest sprouting. Despite significant (*P* < 0.001) environment and genotype-by-environment interactions within and between field and GH experiments, eight genotypes that included five cultivars, two landraces, and one inbred line had consistently high seedling emergence (mean value > 85%) across nine environments. Moreover, 21 environment-specific quantitative trait loci (QTL) were detected in GWAS analysis on chromosomes 1B, 1D, 2B, 3A, 3B, 4A, 4B, 5B, 5D, and 7D, indicating complex genetic inheritance controlling seedling emergence. We aligned QTL for known traits and individual genes onto the reference genome of wheat and identified 16 QTL for seedling emergence in linkage disequilibrium with coleoptile length, width, and cross-sectional area, pre-harvest sprouting and dormancy, germination, seed longevity, and anthocyanin development. Therefore, it appears that seedling emergence is controlled by multifaceted networks of interrelated genes and traits regulated by different environmental cues.

## Introduction

Wheat crop in Western Australia (WA) has traditionally been sown in moist soils at the beginning of the growing season in late autumn (April–May), after which it grows through the cooler months until flowering and subsequently filling grain in spring (September–November) as rainfall declines and temperature rises. However, rainfall and temperature patterns are changing in the agricultural areas of WA. Decreasing rainfall and the increasing temperature have resulted in water-limited potential across Australia declining by 27% from 1990 to 2015 (Hochman et al., 2017), although actual wheat yields in WA have risen slightly since 1990 due to better production technology being developed and adopted over this time. For instance, in this environment wheat yields decline by at least 20 kg/ha each day as sowing is delayed in May and June (Shackley and Anderson, 1995), and there has been a long-term trend for earlier sowing of wheat (Stephens and Lyons, 1998). Simulation modeling suggests further increases in the yield are possible with even earlier sowing in early April or March, but there are no currently available wheat cultivars well adapted to WA conditions with appropriate maturity for such early sowing (Hunt et al., 2019). Climate change related rainfall decline in the WA Wheatbelt has been greatest in May and June when crops are traditionally sown, and there has been an increase in March rainfall (Cai and Cowan, 2013; Hochman et al., 2017; Scanlon and Doncon, 2020). This means that soil moisture availability for crop establishment will often be lower in the traditional sowing window than in the past, but also there will be more frequent sowing opportunities in March and early April than before. These earlier sowing opportunities will be associated with higher temperatures and evaporation rates than in late April and May, so soil water availability is likely to be limiting for crop establishment more often.

A strategy to overcome soil water limitations is to sow into deeper (>10 cm) soil layers less prone to surface evaporation losses, especially if there has been significant summer rain. However, deep sowing of wheat often results in poor emergence and stand establishment, and lower yields in dryland environments (Photiades and Hadjichristodoulou, 1984; Schillinger et al., 1998; Rebetzke et al., 2007). Modern semi-dwarf wheats are particularly sensitive to this because the major genes controlling semi-dwarf stature, *Rht-B1b* and *Rht-D1b*, decreased sensitivity to gibberellins resulting in a significant reduction in cell elongation of vegetative tissues and shorter coleoptiles (Keyes et al., 1989; Botwright et al., 2005). There are alternative dwarfing genes exploiting different hormone pathways that can simultaneously control semi-dwarf plant stature without decreasing coleoptile length. Semi-dwarf genotypes with *Rht8* and *Rht9* dwarfing genes and long coleoptiles have been developed (Rebetzke et al., 1999; Rebetzke and Richards, 2000) maintaining good establishment and high yields following deep sowing (Rebetzke et al., 2007), but there are no commercial cultivars incorporating these genes yet with good adaptation to WA conditions.

Soil moisture is not always available within 10–20 cm of the soil surface at sowing time, and deep sowing has no advantage under these conditions. Wheat sown 5 cm deep or shallower is susceptible to declining soil moisture availability through evaporative losses from the soil surface, and the ability to emerge quickly due to the limiting soil moisture is an important trait for crop establishment. Seed germination, dormancy, and vigor involve the interaction of biochemical, physiological, and molecular mechanisms regulated under different environmental cues, such as low or high temperatures, growth regulators, and differing water regimes (reviewed in Kucera et al., 2005; Weitbrecht et al., 2011; Rajjou et al., 2012). The transition from germination to cell elongation and coleoptile growth in grasses also involves the accumulation of cross-linking cellulose, non-cellulosic polysaccharides, and phenols influenced by different environmental signals (Carpita, 1996; Carpita et al., 2001) exacerbating the complexity of developmental dependent biological mechanisms involved in seedling emergence. It is, therefore, reasonable to assume that germination and coleoptile growth required for emergence is under multifaceted genetic control with significant environmental influences.

Although the coordinated response of biological mechanisms associated with traits controlling seedling emergence is not well defined, numerous studies have reported a plethora of quantitative trait loci (QTL) across most wheat chromosomes controlling seed dormancy, germination, and vigor (reviewed in Gao et al., 2013; Vetch et al., 2019). Similarly, many QTL for coleoptile length, width, and thickness have been identified on chromosomes 1B, 1D, 2B, 3A, 3B, 4A, 4B, 5B, 5D, and 7D (Rebetzke et al., 2007, 2014; Spielmeyer et al., 2007; Li et al., 2017; Ma et al., 2020; Sidhu et al., 2020) and collectively established the polygenic nature of traits likely to be associated with seedling emergence. Moreover, increasing reports of wheat genes expressed at seed germination (Aoki et al., 2006; Nakamura et al., 2011; Liu et al., 2013; Yu et al., 2014; Pearce et al., 2015; Kim et al., 2016; Son et al., 2016; Izydorczyk et al., 2018), during dormancy (Nakamura et al., 2007; Yang et al., 2007; Lei et al., 2013; Gao et al., 2014; Zhang et al., 2014; Torada et al., 2016), and through coleoptile growth under stressed and non-stressed conditions (Singla et al., 2006; Ahmed et al., 2009; Xing et al., 2009; Himi et al., 2011; Shoeva et al., 2014; Shin et al., 2016) confirm the myriad of genes associated with biological mechanisms underpinning seedling emergence. Despite numerous QTL and genes associated with germination and coleoptile growth, it is not known which genomic regions have a significant influence on seedling emergence under limited soil moisture environments.

Notwithstanding the importance of establishing crops in water-limited soils, evaluation for seedling emergence has been reported for a limited number of wheat varieties (Khah et al., 1986; Addae and Pearson, 1992; López-Castaneda et al., 1996; Gooding et al., 2006; Akinci et al., 2008; Buriro et al., 2011). The exception was a study that evaluated a large population of wheat genotypes when 662 lines were sown deep into the soil profile (Mohan et al., 2013). Approximately 28% of the variability for seedling emergence across multiple environments was attributed to coleoptile length while unknown factors accounted for the remaining variation (Mohan et al., 2013). The influence of physical soil constraints may also contribute to variation and a recent study identified differences in the seedling emergence when 38 wheat genotypes were evaluated through soils with a surface crust (Anzooman et al., 2018). To the best of our knowledge, there are no known reports of wheat genotypes evaluated for their ability to germinate and emerge in field environments when water availability in the soil top layer was the limiting factor.

Evaluating wheat genotypes for the genetic potential to germinate and emerge during intermittent rainfall events in early autumn when sown in the soil top-layer (∼4 cm depth) provides an alternative approach for crop establishment under limited water availability than deeper sowing (∼10 cm). Therefore, this study aimed to: (1) evaluate 233 global wheat genotypes for high seedling emergence under limited water availability in the field and controlled environments when sown at 4 cm depth; (2) ascertain genetic loci associated with phenotypic variation for seedling emergence at each environment by genome-wide association study (GWAS) using single nucleotide polymorphic (SNP) markers from the iSelect Infinium 90K genotyping array; and (3) compare known loci and genes controlling variation with seed vigor, dormancy, germination, and coleoptile characteristics, and extrapolate their relationship with seed emergence under limited water availability. This study will provide information on wheat lines suitable as donor parents for breeding improved varieties, and further knowledge on the genetic control and biological mechanisms underpinning traits regulating seedling emergence under limited water conditions in different environments.

## Materials and Methods

### Environment Characterization

Field experiments in 2017–2019 were undertaken at the Merredin Research Station, Department of Primary Industries and Regional Development (DPIRD; formerly Department of Agriculture and Food Western Australia). We set the environmental context for these experiments using data from the automatic weather station at DPIRD’s Merredin Research Station (weather.agric.wa.gov.au/map) between 2001 and 2019. Farmers in WA often plant wheat into dry soil in late April or May, but generally will not plant earlier unless at least 20 mm rain falls over 3 consecutive days. We identified instances when this happened between 1 March and 31 May from 2001 and 2019, and recorded maximum soil temperature at 4 cm depth (seed depth) and pan evaporation [Class A pan simulated by the Penman-Montieth equation (Montieth, 1965)] each day for the next 7 days.

### Plant Material

A population of 233 wheat genotypes was acquired for the evaluation of seedling emergence and coleoptile characteristics and GWAS. The population consisted of 74 commercial varieties, 95 inbred lines, and 64 landraces from different regions of the world (Supplementary Table S1). Inbred lines were accessed through the Centro Internacional de Mejoramiento de Maiz y Trigo (CIMMYT) Australia International Center for Agricultural Research in the Dry Areas (ICARDA) germplasm exchange (CAIGE) project^1^. Commercial varieties and landraces were accessed from the collection at DPIRD and the Australian Grains Genebank (Horsham, VIC, Australia), respectively.

Seeds of all lines were propagated annually in the glasshouse (GH) in 2016 and 2017, and in the field at Merredin Research Station, WA, in 2018. Seeds propagated in each year were used in both field and GH experiments in the subsequent year. The viability of seed harvested in each year was determined using germination tests where 100 seeds from 30 random samples propagated in each year were placed on moist filter paper and incubated at 25°C in the dark for up to 10 days. Representative samples had 95–100% germination and were, therefore, deemed suitable for emergency studies in both fields and GH experiments.

### Design and Settings for Field Experiments

The experiments were sown in dry sandy loam textured topsoils in mid-autumn (12 April 2017, 13 April 2018, and 18 April 2019) and late autumn (10 May 2017, 15 May 2018, and 10 May 2019). Uniform seed beds were prepared in the field by scarification and rolling within 1 week prior to sowing. The plots were arranged in a rectangular array as spatial row-column designs and generated using DiGGer software (Coombes, 2002). Partially replicated randomized experiments (Smith et al., 2006) included 118 non-replicated lines, 101 duplicated lines, and 14 triplicated lines. The experiments were arranged in 12 columns by 30 rows. In April 2017, rows 1–15 and 16–30 were under two different adjacent rain-out shelters whereas experiments in May 2017, April and May in 2018, and 2019 were sown in an open field. A total of 360 plots for each experiment (180 plots in each rain-out shelter in April 2017) were sown as 1 m single rows with 0.4 m between rows and 0.5 m spacing between plots. Each plot contained 50 seeds (visually inspected for uniformity and sieved at > 2.8 mm diameter) and was sown using a single-row mechanical plot drill seeder (Rowseed 1R, Wintersteiger AG, Austria) at a soil depth of 4 cm into a dry seed bed.

Surface drip irrigation (SDI) was installed in all field experiments. The SDI consisted of T-tape model 508-15-220 (Rivulis Irrigation, Israel) having 16 mm tape diameter, 0.22 mm tape thickness, 150 mm spacing between emitters, and a flow rate of 220 L/h per 100 m of tape at 0.55 bar. Tapes were laid on the soil surface perpendicular to the plots spaced 18 cm apart, covering an area of ∼300 m^2^. The experiments were irrigated with 15–20 mm of water immediately after sowing with no further irrigation or rain events in 2017. An additional 10 mm of water was applied to experiments in 2018 and 2019 either as supplementary irrigation or through rain events. Therefore, each experiment had 15–30 mm of water applied. Soil moisture content was monitored in field experiments at 4 cm (seed depth) and 10 cm using frequency domain reflectometry (CS-616 sensors, Campbell Scientific Australia) by deploying 12 pairs of sensors installed between the crop rows, one at each depth. Soil temperature was recorded at 4 cm soil depth at two locations in each experiment. There was also a tipping bucket rain gauge adjacent to each experiment. Data were recorded at hourly intervals using a Campbell CR-10X^®^ data logger. Seedling emergence percentage was monitored by counting individual plants in each single-row plot at 24-h intervals beginning 7 days after initial irrigation. Cumulative seedling emergence was monitored for all genotypes across each experiment until a plateau phase was reached. Percentage seedling emergence for each genotype was calculated at the commencement of the plateau phase.

### Design and Settings for Glasshouse Experiments

Soil (loamy sand with a Munsell Soil Color of 10YR 7/4) was sourced from Merredin Research Station, dried and sifted through a 2 mm sieve to obtain a homogenized soil of consistent particle size. The dried soil was mixed with water to obtain the 9% gravimetric soil moisture (56% plant available water content), placed in a plastic PP Multistaka Crate (22 L, 390 mm × 290 mm × 120 mm; Plasdene, Australia) and leveled within 10 mm from the top of the tub. A total of 72 tubs were used in each experiment and configured as four columns of 18 rows supported by mesh benches in the GH. Each tub consisted of 5 mm × 250 mm single row plots, hence a matrix of 20 × 18 (total of 360 plots) for each experiment. Spatial row-column designs were generated using DiGGer software. A partially replicated randomized experiment was designed and included 118 non-replicated lines, 101 duplicated lines, and 14 triplicated lines distributed across the experiment. Ten seeds of each replicate were sown on the same day in a 25 cm single row plot at 4 cm soil depth.

Gravimetric soil moisture was 0.09 at the beginning of each GH experiment and lids were kept on the tubs throughout to minimize moisture loss. Gravimetric soil moisture at the end of each experiment was measured in 40 of the 72 tubs by weighing a subsample of the soil before and after drying at 105°C. Volumetric moisture content (VMC) was measured in each tub at the same time with a capacitance soil moisture probe (MP406, ICT International, Armidale, Australia). Randomly allocated iButtons (DS1921G, Thermochron Australia) were buried at seed depth in 18 of the 72 tubs and used to record soil temperature at 15 min intervals. Each tub was covered with a white plastic lid to ensure retention of moisture and the number of newly emerged seeds was counted daily for each plot until the cumulative emergence across the matrix plateaued, at which point the experiment was concluded.

### Coleoptile and Shoot Measurements in Controlled Environments

To account for potential spatial variability in the controlled environment, a row-column design with two replicates for each genotype was used for conducting the experiment. Good quality seeds free of any visible damage or shriveling were sourced for all lines and then individually sized to a range of 40–45 mg. Seeds were sown in deep, wooden seedling trays [dimensions 600 mm (L) × 300 mm (W) × 120 mm(D)] containing a fertile, compost-based potting mix, and at a sowing depth of *ca.* 2 cm below the soil surface. Each tray was watered thoroughly and allowed to drain before covering with a lid and securing with a large opaque plastic bag to exclude light. Trays were placed into darkened growth cabinets set at a constant soil temperature of 15°C. This temperature was chosen to represent soil temperatures commonly encountered throughout Australian and other global cropping zones at sowing (Rebetzke et al., 2014). Trays were left until 200°C (assuming a base temperature of 0°C) whereupon they were removed for assessment. Coleoptile lengths were determined with a ruler as the distance from the scutellum to the tip of the coleoptile. Coleoptile width and thickness were determined midway along the coleoptile for all lines following the methodology of Rebetzke et al. (2004). Briefly, coleoptile width was assessed as the distance across the coleoptile at the widest axis and then perpendicular to this axis. A digital micrometer was used for all coleoptile diameter measurements.

### Statistical Analyses

All the statistical analyses were conducted using GenStat (VSN International, 2020). The linear mixed models were used for the analyses of all experiments. Each experiment was analyzed separately, where the statistical model accounted for the experimental design (plot structure), the present spatial variability and for the genotype effect and, where present, covariates (the treatment structure). All plot structure components were fitted as random terms, while the treatment structure terms were fitted as fixed or random depending on the objectives. Fitting genotype as fixed terms allowed comparison based on their means, obtained as best linear unbiased estimates (BLUEs). Best linear unbiased predictors (BLUP) of means in the controlled environments were obtained by fitting genotype as the random term along with spatial adjustments, such as the AR1xAR1 to account for the presence of a local trend and linear row or/and column effects to account for global trends. The tests for normality and homogeneity of the error variance indicated that there was no need for applying generalized linear mixed models for the response variable. The selection of the final model was based on the use of the Akaike information coefficient (AIC) and the log likelihood ratio tests. Meta-analysis and variance components analysis were applied to assess similarities in genotype rankings between environments and to assess the proportions of the total phenotypic variance due to the environment, the genotypes, and their interaction. Additionally, broad sense heritability was obtained as the ratio of genetic variance to total phenotypic variance.

### Genotyping

DNA samples from wheat accessions were assayed using the 90K Infinium SNP chip array (Wang et al., 2014). Raw intensity data for each SNP was extracted and analyzed in GenomeStudio, NormTheta, and NormR values. A custom perl script was used to cluster samples and assign genotypes to known polymorphisms. For genome-wide association tests, monomorphic markers, those with less than 80% call rate, and minor allele frequencies (MAF) less than 5% were removed from the dataset. A total of 19,745 SNP markers were used for statistical analysis. The physical location of SNP markers was extracted from Pretzel *via* https://plantinformatics.io/ (Keeble-Gagnère et al., 2019) using the International Wheat Genome Sequencing Consortium (IWGSC) RefSeq v1.0 physical map (Alaux et al., 2018).

### Linkage Disequilibrium Decay, Genome-Wide Association, and Statistical Analysis

A TASSEL v.5.2.52 was used to identify marker-trait associations (MTAs) (Bradbury et al., 2007). A mixed linear model (MLM) was used to account for both population structure and cryptic relatedness (Q + K). The genotypic kinship matrix (K) was estimated by selecting the “Centered_IBS” method and population structure (Q) was corrected using the principal component (PC) analysis. The suitable number of PCs for each trait was determined by testing one through 15 PCs with visual assessment of quantile-quantile plots (Q-Q plots). The option “P3D” was not selected during the MLM analysis with the variance component re-estimated after each marker. The R programs ‘qqman’ and ‘Rcolorbrewer’ were used to draw Manhattan plots (Turner, 2017; R Core Team, 2018). Two-dimensional displays of the top PCs were drawn in R.

A genome-wide significance threshold for MTAs was set at − log_10_ (*p*) > 5.61 using Bonferroni correction with α = 0.05. Bonferroni correction is highly conservative and reduces type I errors and is most practical when all tests are independent (Bland and Altman, 1995; Abdi, 2007). To estimate the number of independent tests, the tagger function in Haploview was implemented as described in Maccaferri et al. (2016) with an *r*^2^ of 0.1. This returned a genome-wide threshold significance of − log_10_ (*p*) > 4.11 and was considered a moderate level of significance compared with the Bonferroni corrected threshold. As reported in several related studies, a threshold of significance of − log_10_ (*p*) > 3.00 was included as a suggestive level of significance (Maccaferri et al., 2015; Alomari et al., 2017; Muqaddasi et al., 2019).

Linkage disequilibrium (LD) was assessed to identify MTAs that occurred as single entities or clusters of SNPs. The “–block” function in PLINK 1.9 was utilized to identify intra-chromosomal SNPs in strong LD as defined in Gabriel et al. (2002) with the bottom of the 90% D-prime *CI* greater than 0.70 and the top of the *CI* at least 0.98 (Purcell et al., 2007). LD decay was estimated to gauge the approximate size of QTL blocks. Marker pairwise *r*^2^ values were calculated in PLINK 1.9 with a sliding window of 50 and then LD decay curves fitted by non-linear regression for each sub-genome (A, B, and D) as described by Marroni et al. (2011) with the decay of *r*^2^ against distance. LD decay plots were drawn in R with a critical threshold of *r*^2^ = 0.2 (R Core Team, 2018).

### Alignment of Quantitative Trait Loci and Candidate Genes to the Physical Map of Bread Wheat

Previously identified QTL controlling traits for pre-harvest sprouting (Albrecht et al., 2015; Lin et al., 2016; Martinez et al., 2018; Zhu et al., 2019), germination (Tarawneh et al., 2019), seed longevity (Arif and Börner, 2020), coleoptile characteristics (Rebetzke et al., 2014; Li et al., 2017; Ma et al., 2020; Sidhu et al., 2020), and grain color (Lin et al., 2016) were physically anchored to the Infinium 90K array using marker information available in Pretzel (Keeble-Gagnère et al., 2019^2^). Markers that could not be anchored using Pretzel, were putatively anchored to the IWGSC RefSeq v1.0 physical map (Alaux et al., 2018) using the BLAST tool at URGI INRA^3^. Physical locations of genes controlling germination (Aoki et al., 2002; Appleford et al., 2006; Nakamura et al., 2011; Lei et al., 2013; Liu et al., 2013; Yu et al., 2014, 2016; Pearce et al., 2015; Kim et al., 2016; Izydorczyk et al., 2018), coleoptile characteristics (Singla et al., 2006; Himi et al., 2011; Strygina and Khlestkina, 2019), grain color (Himi et al., 2005, 2011; Shoeva et al., 2014), dormancy, and pre-harvest sprouting (Nakamura et al., 2007; Yang et al., 2007; Gao et al., 2014; Zhang et al., 2014; Son et al., 2016; Torada et al., 2016; Yu et al., 2020) were also determined using the BLAST tool at URGI INRA, requiring a minimum 95% identity with the associated GenBank accession to anchor to the physical map.

## Results

### Environment Characterization in 2001–2019

**FIGURE 1:**
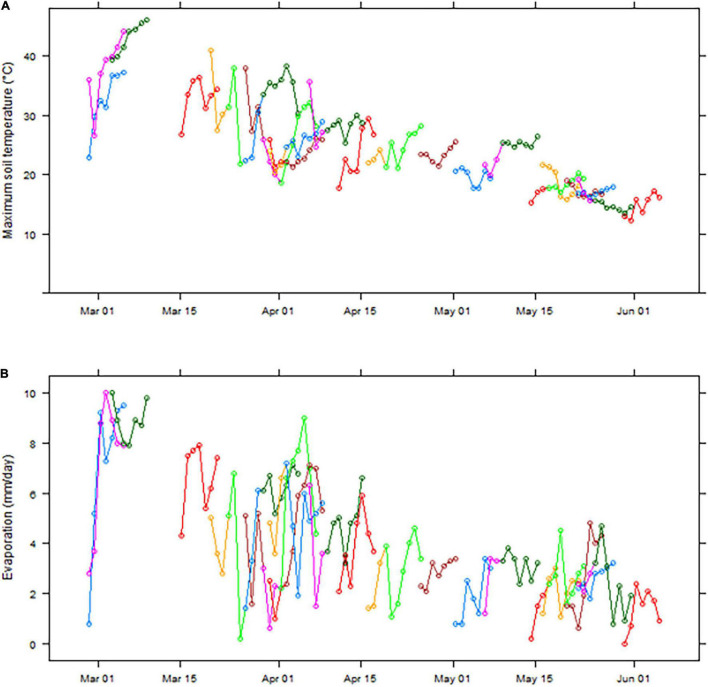
Maximum soil temperature at 4 cm **(A)** and daily evaporation (simulated Class A pan) **(B)** in the 7 days following sowing opportunities (defined as occurring after a period of 3 days with ≥ 20 mm rainfall) at Merredin, Western Australia, from March to May in the years 2001–2019. Separate colored lines represent individual sowing opportunities within the data set, e.g., the first blue line in each panel shows data for a sowing opportunity on 28 February 2006, and the first pink line on 28 February 2015.

Figure 1 shows how soil temperature at normal sowing depth and potential evaporation rates following sowing opportunities at Merredin decline from March through to June. During the week following a sowing opportunity (defined as occurring when there has been ≥ 20 mm rain in the previous 3 days) in March and the first half of April, soil temperatures are often above 30°C, and sometimes above 45° (Figure 1A). However, after the middle of April, soil temperatures are much cooler and only occasionally exceed 25°C (Figure 1A). Moreover, evaporation is typically low on the 1st day following a sowing opportunity preceded by 20 mm rain, then rises to between 6 and 10 mm/day for March and early April but is usually less than 4 mm/day after the middle of April (Figure 1B). Therefore, analysis of current and historical measurements indicated that lower soil temperature and evaporation rate from mid-April onward in any given season provided suitable sowing opportunities to evaluate the percentage seedling emergence under limited water conditions.

### Soil Moisture and Temperature in Field Experiments

**FIGURE 2:**
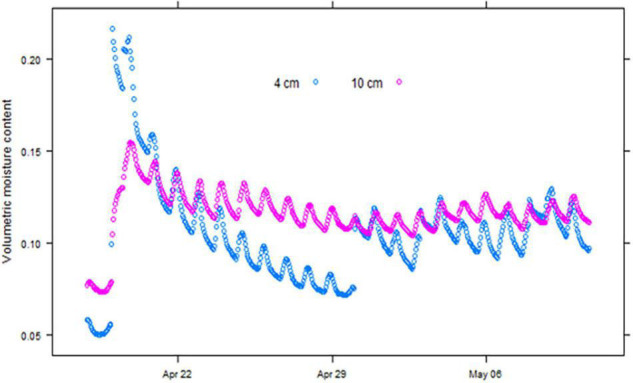
Soil moisture recorded at hourly intervals at 4 cm and 10 cm depth in the April 2019 wheat emergence experiment at Merredin, Western Australia.

The soil moisture content at 4 cm responded within an hour of irrigation being applied and reached its peak within 2 h, after which it declined steeply through drainage and surface evaporation. A typical soil moisture recording for the duration of a trial conducted in April 2019 is shown in Figure 2. The moisture content at 10 cm took longer to reach its peak but was more stable than close to the surface. Changes in the total amount of water in the top 15 cm of the soil calculated, assuming the average of the moisture content at 4 cm and 10 cm are good estimates of the average moisture content over the top 15 cm of the soil profile, and closely match the amounts of applied irrigation and rainfall.

The soil temperature and moisture content were monitored during each field experiment. The soil on which field experiments were conducted in 2017–2019 was loamy sand to sandy loam texture with a drained upper limit around 0.12 VMC and a lower limit around 0.06 VMC. The moisture content at seed depth remained above the lower limit during the first 6 days of each experiment when the germinated seedlings had limited access to deeper soil moisture, but it did get below 20% plant available water capacity (PAWC) at this depth in April 2017 (Table 1). Soil moisture at seed depth did not fall below 50% PAWC in any of the May experiments. The soil was consistently warmer in the April experiments, with the temperature rising above 30°C in each year, but no extreme temperatures were recorded in any May experiment (Table 1).

**TABLE 1:** Average and minimum volumetric soil moisture contents (VMC, cm^3^cm^–3^) recorded at 4 and 10 cm depth, and average and maximum soil temperature at 4 cm depth, in the first 6 days after sowing six field experiments conducted at Merredin, Western Australia.

Experiment	VMC 4 cm		VMC 10 cm		Soil temperature (°C)	
	Average	Min	Average	Min	Average	Max
April 2017	0.107	0.070	0.121	0.081	24.1	32.0
May 2017	0.136	0.104	0.119	0.100	18.2	26.4
April 2018	0.118	0.087	0.116	0.099	21.5	33.2
May 2018	0.132	0.101	0.123	0.099	17.2	24.9
April 2019	0.113	0.077	0.109	0.081	18.8	34.4
May 2019	0.146	0.095	0.139	0.111	17.4	26.1

### Soil Moisture and Temperature in Glasshouse Experiments

The gravimetric moisture content of the soil used in the GH experiments at drained upper limit (ψ_*M*_ = − 10 kPa) and lower limit (ψ_*M*_ = − 1500 kPa) was 0.119 and 0.053, respectively. The initial moisture content of 0.09 was, therefore, 56% of PAWC. This changed very little over the duration of each experiment (Table 2). Gravimetric moisture was almost identical at the end of each experiment, but volumetric moisture was higher after the end of the 2019 experiment, suggesting that the soil was packed to a higher bulk density, than in the two 2018 experiments. Soil temperature fluctuated significantly throughout these experiments, increasing to 30°C on several occasions in the two June experiments. The soil was cooler in the July 2018 experiment than either June experiment but still reached 30°C on one occasion (Table 2).

**TABLE 2:** Gravimetric and volumetric moisture contents (GMC and VMC, respectively) at the completion of the monitoring period, and average, maximum, and minimum soil temperatures during the monitoring period in three glasshouse (GH) experiments, conducted at Merredin, Western Australia.

Experiment	GMC	VMC	Soil temperature (°C)		
			Average	Max	Min
June 2018	0.083	0.097	16.9	32.5	8.5
July 2018	0.084	0.099	15.0	30.0	5.0
June 2019	0.084	0.112	17.5	35.0	8.5

### Phenotypic Analysis of Seedling Emergence

The histograms of predicted emergence values and Shapiro–Wilk tests showed non-normal distribution in each environment, supported by moderate skewness and kurtosis (Supplementary Figure S1). Variable population means and median values (41.9–95.8% and 47.1–99.5%, respectively) were evident between field and GH environments (Table 3). Similarly, broad-sense heritability was variable from low in the field trial in April 2018 (*H*^2^ = 0.15 ± 0.09) to high (*H*^2^ = 0.73 ± 0.04) in the GH experiment in June 2019 (Table 3) indicating the varying proportion of phenotypic variance attributed to genetic differences within each environment.

**TABLE 3:** Summary statistics of percentage field seedling emergence for 233 wheat lines in April (04) and May (05) in years 2017–2019 and GH seedling emergence in June (06) and July (07) in years 2018–2019.

	Field 0417	Field 0517	Field 0418	Field 0518	Field 0419	Field 0519	GH 0618	GH 0718	GH 0619
Minimum	26.9	6.9	0.0	14.0	2.0	0.0	60.0	7.1	0.0
Maximum	100.0	98.6	100.0	100.0	98.1	100.0	100.0	100.0	100.0
Mean	74.4	80.7	63.2	77.9	54.2	45.7	95.8	79.8	41.9
Median	76.4	83.8	63.9	81.0	56.5	47.1	99.5	85.4	39.0
S.E.D.	16.9	7.6	19.9	12.3	17.2	14.7	6.7	16.6	14.4
*H* ^2^	0.34 ± 0.08	0.48 ± 0.06	0.15 ± 0.09	0.54 ± 0.05	0.57 ± 0.05	0.61 ± 0.04	0.39 ± 0.08	0.46 ± 0.05	0.73 ± 0.04

Spearman’s rank-order co-efficient (ρ) between environments was either not significant (*P* > 0.05) or significant (*P* < 0.05) but low between the environments (Table 4). The exception was between the experiments evaluated in two field and one GH environment in 2019 where ρ = 0.67–0.73 (*P* < 0.001) (Table 4). It appears, therefore, that the percentage emergence of genotypes was variable across environments particularly between years, an inference confirmed by visual comparison of frequency distributions (Supplementary Figure S1). Subsequent meta-analysis for seedling emergence across field and GH environments showed that each term or interaction was highly significant (*P* < 0.001) in contributing to the total phenotypic variation in both the field and GH conditions (Table 5). The largest estimated proportion of the total phenotypic variation evaluated in either field, GH, or both was contributed by environment (36.0–54.8%) followed by genotype × environment (27.5–41.8%) with genotype contributing the smallest (16.3–22.2%) (Table 5).

**TABLE 4:** Spearman’s rank correlation coefficient between environments of seedling emergence for 233 wheat lines evaluated in the field April (04) and May (05) in years 2017–2019 and evaluated in GH in June (06) and July (07) in years 2018 and 2019.

	Field 0417	Field 0517	Field 0418	Field 0518	Field 0419	Field 0519	GH 0618	GH 0718	GH 0619
Field 0417	–								
Field 0517	0.36**	–							
Field 0418	0.11^NS^	0.06^NS^	–						
Field 0518	0.02^NS^	0.08^NS^	0.33**	–					
Field 0419	0.20*	0.12^NS^	0.12^NS^	0.21**	–				
Field 0519	0.14*	0.08^NS^	0.14*	0.18*	0.73**	–			
GH 0618	0.13^NS^	-0.07^NS^	0.08^NS^	0.15*	-0.10^NS^	-0.07^NS^	-		
GH 0718	0.11*^NS^*	-0.02*^NS^*	0.27**	0.46**	0.11^NS^	0.11^NS^	0.14*	–	
GH 0619	0.20*	0.12^NS^	0.23**	0.18*	0.68**	0.67**	-0.10^NS^	0.18*	–

**P < 0.001, *P < 0.05; NS, not significant (P > 0.05).

**TABLE 5:** Meta-analysis for seedling emergence of wheat lines (*n* = 233) for six fields and three GH environments in 2017–2019.

Experiment	Source of variation	Wald statistic	d.f.	*P* *^a^*	Variation (%)*^b^*
Field 2017-2019	Genotype (G)	138866.9	232	<0.001	22.2
	Environment (E)	224970.7	5	<0.001	36.0
	G × E	261702.3	1139	<0.001	41.8
Glasshouse 2018-2019	Genotype (G)	113082.4	232	<0.001	17.7
	Environment (E)	350032	2	<0.001	54.8
	G × E	175905.9	457	<0.001	27.5
Field + Glasshouse 2017-2019	Genotype (G)	209228.12	232	<0.001	16.3
	Environment (E)	596144.28	8	<0.001	46.4
	G × E	479054.06	1828	<0.001	37.3

*^a^F-test probability of Wald statistic.*

*^b^Fraction of Wald statistic associated with each term or interactions.*

Despite having either no or low phenotypic correlation (Table 4) and highly significant (*P* < 0.001) environmental and genotype × environmental interactions (Table 5), nine lines with consistently low (<50%) and eight lines with consistently high (>85%) seedling emergence were identified from the mean values of all field and GH environments (Table 6). Given no common parentage amongst the wheat cultivars with high mean seedling emergence across environments (Supplementary Table S1), it was reasonable to assume that variation contributed by genotypes was derived from different lineages. Interestingly, only two landraces originating from Pakistan (010HAT10 and 013HAT10) were identified as having consistently high seedling emergence (Table 6). On the other hand, a majority of low emerging lines were landraces (Table 6), despite their origin from predominantly dry regions of South West Asia, Asia, Middle East, and North Africa (Supplementary Table S1).

**TABLE 6:** Selected wheat lines with low (<50%) and high (>85%) means for percentage seedling emergence across six fields and three GH environments.

		Percentage seedling emergence									
		Field0417	Field0517	Field0418	Field0518	Field0419	Field0519	GH0618	GH0718	GH0619	Mean
Low emergence	ZVS09Qno33	33	19	43	56	8	0	60	84	0	33.7
	139 HAT10	67	78	25	14	13	11	80	67	17	41.3
	029 HAT10	64	69	28	59	27	7	90	40	7	43.4
	107 HAT10	63	48	51	42	44	38	97	14	0	44.1
	ZWW10Qno155	63	69	41	59	2	0	100	76	0	45.6
	154 HAT10	70	99	39	42	22	30	99	10	14	47.2
	Sunvex	65	58	47	30	32	28	99	37	43	48.8
	116:ZIZ12	41	33	44	71	26	28	100	85	15	49.2
	025 HAT10	58	78	56	88	25	6	100	30	6	49.7
High emergence	013 HAT10	85	94	86	92	70	64	100	100	79	85.6
	Yandanooka	86	88	75	70	87	77	99	100	86	85.3
	Chino466	95	86	81	94	90	60	99	86	79	85.6
	Frame	91	92	81	98	76	67	100	97	75	86.3
	157:ZIZ13	87	74	63	92	89	86	100	100	88	86.6
	Hartog	93	91	87	93	88	57	99	90	100	88.7
	Espada	95	95	89	86	83	80	81	89	100	88.7
	010 HAT10	77	96	90	92	80	92	100	97	92	90.7

*Percentage seedling emergence for individual environments are indicated in April (04), May (05), June (06), and July (07) in years 2017–2019.*

### Phenotypic Analysis of Coleoptile Length and Width

Large and statistically significant (*P* < 0.05) differences between individuals of the population were observed for coleoptile length cross-sectional area and shoot length (Table 7). Similarly, repeatabilities were high on an entry-mean basis (*R*^2^ = 0.62–0.76, *P* < 0.01) for all three characters, indicating confidence in the among-entry comparisons. The landraces 066HAT10 and 087HAT10 were the longest for coleoptile and shoot lengths, respectively, while the ICARDA entry 96:ZIZ13 was the largest for cross-sectional area (Table 7). Similarly, ICARDA entries 47:ZIZ13 and 13:ZIZ13 were the shortest for coleoptile and shoot length, respectively, whereas the landrace 048HAT10 had the smallest coleoptile cross-section area (Table 7). The Australian and CIMMYT entries together with the remaining Australian varieties and CIMMYT inbred lines had intermediate values for coleoptile and shoot length and cross-section area.

**TABLE 7:** Summary statistics of coleoptile length and cross-sectional area and shoot length for 233 wheat lines evaluated in controlled environmental conditions.

	Coleoptile length (mm)	Coleoptile cross-sectional area (mm^2^)	Shoot length (mm)
Minimum	24 (47:ZIZ13)	0.54 (048HAT10)	21 (13:ZIZ13)
Maximum	205 (066HAT10)	3.06 (96:ZIZ13)	282 (087HAT10)
Mean	91	1.76	227
LSD (Between entries)	23	0.51	73
*H* ^2^	0.71 ± 0.05	0.62 ± 0.07	0.76 ± 0.06

*Entries for maximum and minimal values are shown in parentheses.*

### Genetic Relatedness and Linkage Disequilibrium

**FIGURE 3:**
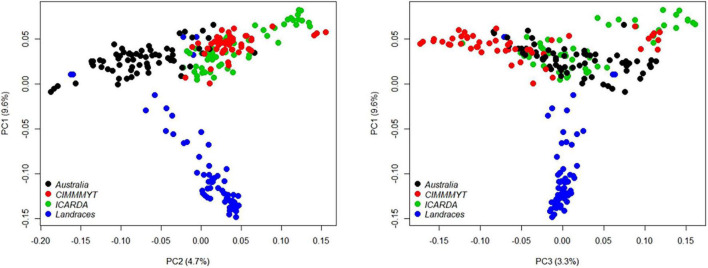
Principal component analysis (PCA) of 233 wheat genotypes from Australia (black), CIMMYT (red), ICARDA (green), and global landraces (blue). Principal components (PCs) were determined using 19,745 SNP markers filtered from the 90K Infinium SNP chip array.

**FIGURE 4:**
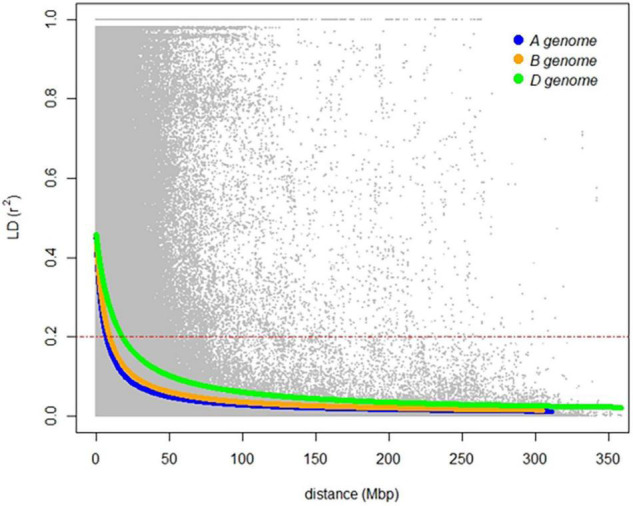
Linkage disequilibrium of the genome-wide association study (GWAS) panel for the A (blue), B (orange), and D (green) sub-genomes of hexaploid wheat based on 19,745 SNP markers. A red dashed line indicates linkage decay level (*r*^2^ = 0.2) of physical distance (in Mbp).

The genetic relatedness of the GWAS panel was evaluated using 19,745 SNP markers based on the principal component analysis (PCA). The first three PCs indicated that 17.6% of the total genetic variance was accounted in the first three PCs (PC1 = 9.6%, PC2 = 4.7%, and PC3 = 3.3%, respectively) (Figure 3). Landraces had a distinct grouping from other lines from breeding programs originating from different continents (Figure 3). The genetic relationship between Australian cultivars and inbred lines from CIMMYT and ICARDA was distinct in the first two PCs with close relatedness and presumably similar historical lineages (Figure 3). LD for 19,745 SNP loci was estimated based on the pairwise squared correlation co-efficient with the threshold of *R*^2^ = 0.2. LD decay was estimated at 6.84 and 8.98 Mbp for the A and B sub-genomes but extended to 17.75 Mbp for the D sub-genome (Figure 4).

### Marker-Trait Associations for Seedling Emergence

**FIGURE 5:**
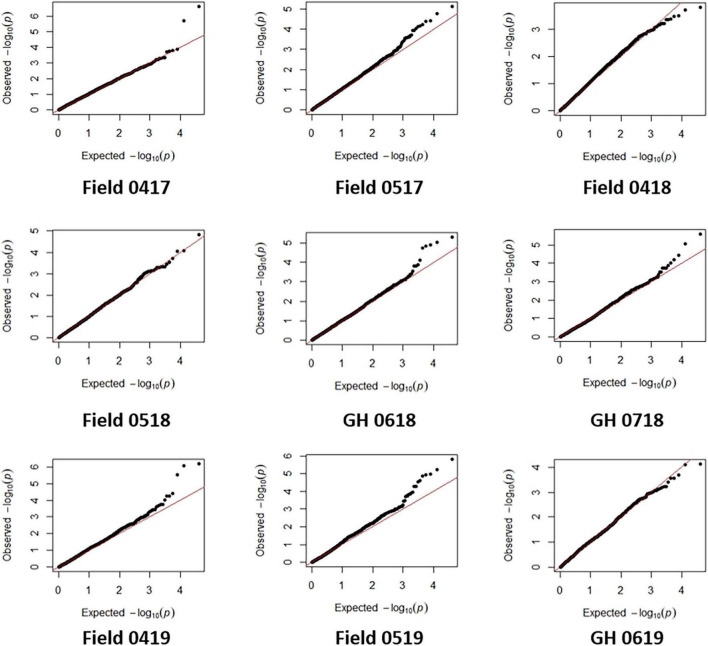
The quantile-quantile (Q-Q) plots for seedling emergence showing observed associations compared with the expected statistical association under the null hypothesis of no association for six fields and three glasshouse (GH) environments in 2017–2019.

An MLM was applied and account for population structure and cryptic relatedness and thereby reduce the rate of false-positive marker-trait associations. BLUE for seedling emergence from each field and GH environment were analyzed using 19,745 SNP markers. Deviations of the observed association compared with the statistics expected for the null hypothesis for most environments were inferred by visual assessment of Q-Q plots (Figure 5) indicating that variation in SNP markers was associated with the seedling emergence in most environments. The exceptions were for field 0418 and 0518 and GH 0619 environments whereby Q-Q plots (Figure 5) indicated either few or no association with SNP markers.

**FIGURE 6:**
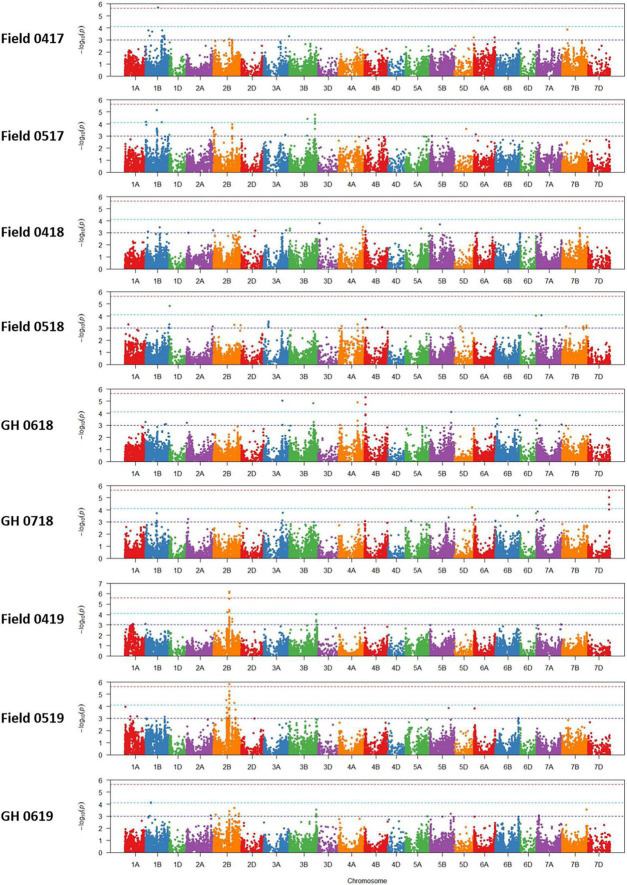
Manhattan plots of marker-trait associations for seedling emergence under six field and three GH environments in 2017–2019.

**FIGURE 7:**
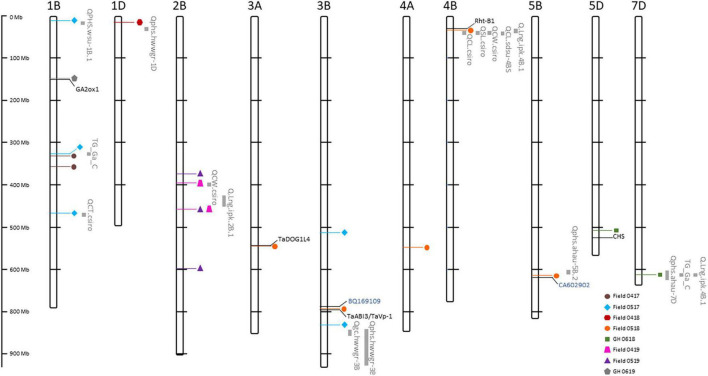
Physical map position of quantitative trait loci (QTL) controlling seedling emergence from eight of the nine Western Australian environments in 2017–2019. QTL from each environment are represented by color coded shapes. Known QTL for traits associated with coleoptiles and shoot characteristics, pre-harvest sprouting, germination percentage and ratio, grain color, and seed longevity (Supplementary Table S3) are shown in gray. Known genes for hormone regulation and seed germination are denoted in black, whereas expressed sequence tags (EST) in seed germination are denoted in blue. Physical distance (Mbp) of chromosomes are shown to the left of figure.

Association tests showed MTA with at least moderate levels of significance (− log10(*P*) > 4.11) for most environments, indicated by Manhattan plots in Figure 6. The exception was a field environment in April 2018 (Field 0418) where no MTA was detected for at least a moderate level of significance (Figure 6) and concurs with low heritability (*H*^2^ = 0.15) of seedling emergence (Table 3) and Q-Q plots for this environment (Figure 5). A total of 37 SNP markers associated with 21 QTL for seedling emergence were detected on 10 chromosomes from the remaining field and GH environments (Table 8). Markers in LD based on estimated linkage decay values of 8.98 and 17.75 Mbp for the B and D genomes, respectively, were considered as representing a single QTL on chromosomes 1B, 2B, 3B, and 4B (Table 8). The physical map position of the 21 QTL controlling emergence from eight of the nine environments is summarized in Figure 7. The majority of QTL were in linkage equilibrium with physical positions exceeding the linkage decay values of 8.98 Mbp for the B sub-genome and 17.75 Mbp for the D sub-genome (Table 8) and, hence, identified as environment-specific. In some instances, the same QTL was detected across environments, such as *QSe.daw.1B-3* on chromosome 1B and *Qse.daw.2B-3* on chromosome 2B (Figure 7 and Table 8). The estimated allele effect size of SNP was variable, ranging from 6.31% to 27.85% of the average phenotypic values (Table 8). Interestingly, minor allele frequency of < 10% for SNP on chromosome 5D (*IWB15109*) and 7D (*IWB34125*; *IWB34126*; and *IWB30203*) in 0718 GH environment had the largest estimated allele effects of all SNP markers across all environments (Table 8). Therefore, it appears that the genetic control of seedling emergence in some environments is underpinned by gene variants having a significant impact on phenotypic effects.

**TABLE 8:** Single nucleotide polymorphic marker associations identifying QTL for seedling emergence (*QSe.daw*) across six fields and three GH environments in WA.

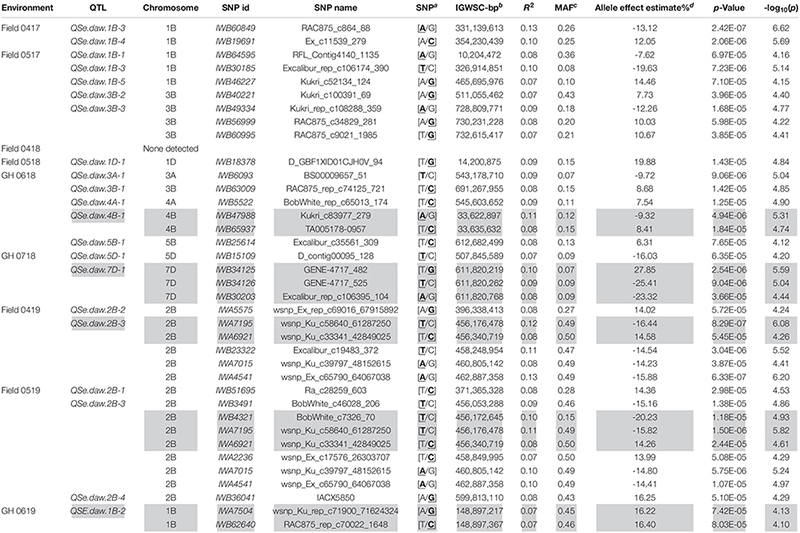

*Quantitative trait loci are above the moderate level of significance (− log10 (p) > 4.11). Gray shading represents makers in strong LD as defined by Gabriel et al. (2002).*

*^a^Desirable SNP for increased seedling emergence based on effect estimate is in bold and underlined.*

*^b^IWGSC RefSeq v1.0, bp: base pairs.*

*^c^MAF: minor allele frequency.*

*^d^The effect estimates the difference between the average phenotypic values of the homozygous A genotype relative to the homozygous B genotype.*

### Comparison of Quantitative Trait Loci for Seedling Emergence With Coleoptile and Shoot Characteristics

The 233 wheat lines evaluated for shoot length, coleoptile length, and coleoptile cross-sectional area in a controlled environment were used in GWAS analysis for QTL detection (*QSL.daw*, *QCL.daw*, and *QCSA.daw*, respectively). A total of eight QTL were detected across all traits that including one QTL for *QCSA.daw*, three for *QCL.daw*, and four for *QSL.daw* (Supplementary Table S2). The *QCSA.daw* did not coincide with QTL for either of the remaining traits but the SNP marker on 1B (*IWB74039*) and 4B (*IWB70672*) detected common QTL for *QCL.daw* and *QSL.daw* (Supplementary Table S2). Therefore, it appears that a common gene controls coleoptile and shoot length when the GWAS population was evaluated in a controlled environment. However, only one SNP marker (*IWB26466*) identifying a QTL for coleoptile length (*QCL.daw.1B-1*) on chromosome 1B (Supplementary Table S2) was in LD with a QTL for seedling emergence, *QSe.daw.1B-1* (Table 8) indicates that the remaining loci controlling coleoptile characteristics did not influence the remaining 20 QTL detected in the field and GH environments.

### Alignment of Known Quantitative Trait Loci and Candidate Gene Mapping in Linkage Disequilibrium With Seedling Emergence

The potential role of traits associated with the control of seedling emergence was further investigated by comparing the physical position of QTL detected in eight of the nine environments (Table 8) with the assignment of previously reported markers and/or genes controlling the coleoptile characteristics (length, width, and thickness), shoot length, pre-harvest sprouting, total germination percentage and ratio, grain color, and seed longevity (Supplementary Table S3) to the IWGSC reference genome. Markers associated with 251 reported QTL for nine traits were anchored to the physical map (Supplementary Table S3) with 143 QTL reported to be located across the 10 chromosomes where QTL for seedling emergence were detected (Supplementary Figure S2). At least one QTL associated with each trait (except germination ratio) was in LD for 17 of the 25 detected QTL controlling emergence (Figure 7). Therefore, genomic regions controlling traits for dormancy related to pre-harvest sprouting, germination, coleoptile characteristics, and seed longevity may also influence variation for emergence phenotype when evaluated in the field environments.

Similarly, several reported studies identified wheat genes associated with various biological mechanisms involved in seed germination, coleoptile development, grain dormancy, and pre-harvest sprouting. A total of 218 reported genes, therefore, were positioned on the physical map of wheat (Supplementary Table S3) where 102 resided on the same chromosomes as QTL controlling emergence (Supplementary Figure S2). A total of six genes were in LD for QTL controlling emergence on chromosomes 1B, 3A, 3B, 5B, and 5D (Figure 7). Included in this study were *GA2ox1* involved in hormone signaling during germination on chromosome 1, *TaDOG1L4* on 3A known to regulate the seed dormancy during germination, and both *TaABI3* and *TaVp-1* (*viviparous*) on 3B functioning in hormone regulation during germination and dormancy, respectively (Figure 7 and Supplementary Table S3). The remaining gene sequences represented by expressed sequence tags (EST) BQ169109 on 3B, CA602902 on 5B (Figure 7) were involved in the hormone regulation during germination (Supplementary Table S3).

## Discussion

High seedling emergence is critical for the successful establishment of wheat crops, particularly under limited soil water availability. Environmental effects can have a significant influence on percentage emergence for crop establishment. Soil temperature and soil water content have major effects on seedling emergence but can only be managed indirectly in the field in dryland cropping systems, whereas other influences, such as soil fertility, compaction, tillage, and surface residue can be more directly controlled (Forcella et al., 2000). Furthermore, earlier crop establishment, which is becoming more common in WA, and climate change are likely to lead to more frequent high temperature and sub-optimal soil moisture during crop establishment in this environment. Using genotypes with better adaptation to these conditions is one solution to the problem. This study, therefore, evaluated a collection of wheat genotypes sourced from different regions of the world and identified genotypes with consistently high percentage seedling emergence under minimal soil water availability, managed across successive years in the field and GH locations. In addition, we have detected loci controlling seedling emergence across all years, many of which are environment-specific. In some instances, the position of QTL aligned with known genomic regions controlling for pre-harvest sprouting, germination, coleoptile characteristics, and seed longevity providing knowledge of individual traits that may affect the seedling emergence under limited water availability in different field conditions.

Analysis of current and historical data indicated that cooler soil temperature from mid-April onward in the eastern WA Wheatbelt since 2001 often did not exceed 25°C. It was reported that germination and seedling emergence of wheat is optimal between 20°C and 30°C (Singh and Dhaliwal, 1972; Addae and Pearson, 1992; Buriro et al., 2011). In particular, seed sown in loamy sand when soil temperatures were in the range of 35–45°C showed an exponential decline (85–0%) in seedling emergence (Singh and Dhaliwal, 1972). The average field soil temperatures in this study (17.2–24.1°C), therefore, showed that experiments conducted from mid-April in each year were optimal for measuring the percentage seedling emergence. Similarly, soil temperatures in GH experiments were within the acceptable limits for the evaluation of seedling emergence. Irrigation simulated a rainfall event of 15–30 mm on dry soils in each experiment where soil moisture, measured as VMC was 0.097–0.146 cm^3^ cm^–3^ at 4 cm depth, which is typical for loamy sand topsoils (<10 cm) previously recorded at in the field at the Merredin Research Station at the peak of the growing season in August and above the minimal amount representative in late spring and summer (Russell, 2005).

The proportion of phenotypic variance for seedling emergence due to genotype was disparate in each field and the GH experiment indicated by variable broad-sense heritability estimates. Heritability estimates have previously been reported to vary between genotypes for some species, such as flax (Saeidi, 2012), so it is assumed that environmental interactions can have a profound effect on the seedling emergence in wheat [e.g., soil temperatures (Rebetzke et al., 2016)]. Phenotypic correlation within and between experiments was generally low (*P* < 0.001) or not significant (*P* > 0.05), with a high proportion of significant (*P* < 0.001) environment and genotype × environment interactions confirming the interplay of genotype and environments on expression seedling emergence. Despite volumetric water content and soil temperature being consistent for at least April and May sowings in the successive years and GH experiments, other environmental variables may have a significant influence on phenotypic responses. Variables, such as the interface of soil burial between genotypes with variable seed size (i.e., large- or small-seeded genotypes), inconsistent light requirements related to germination sensitivity (far-red to red ratio), or air quality in the soil, especially the ratio of oxygen, carbon dioxide and water vapor (Forcella et al., 2000) may affect the seedling emergence across environments. Moreover, factors, such as mechanisms for water transport during soil-seed contact (Wuest et al., 1999), soil compaction or smearing (Nasr and Selles, 1995), and surface crusting (Belnap et al., 2001) cannot be excluded as environmental variables influencing water imbibition, dormancy, germination, and the coleoptile elongation required for seedling emergence. However, in some instances, moderate to high heritability and phenotypic correlations were observed, such as that seen between field and GH experiments in 2019. There is increasing evidence that seed produced in a particular maternal environment may develop biological signals that directly interact with zygotic tissues affecting germination, coleoptile characteristics, and other seed properties of the progeny (Penfield and MacGregor, 2017). The seed source for 2019 experiments was from the mother plants grown in field plots at Merredin Research Station in 2018 rather than GH grown seed for experiments in 2017 and 2018, so it is plausible that seed grown in the field developed the necessary signal transduction pathways to control seedling emergence of the progeny when sown in a similar environment. Genotypes from Australia (Yandanooka, Frame, Hartog, and Espada), ICARDA (157:ZIZ13), CIMMYT (Chino466), and landraces (010HAT10 and 013HAT10) consistently expressed higher seedling emergence under minimal water availability across field and GH experiments, regardless of the location from where maternal sources were grown and appeared to have cumulative effects of genetic components less affected by environmental variables. Not only will these serve as parental material for developing cultivars in wheat breeding but also as useful genotypes to increase our knowledge regarding the underlying biological mechanisms responsible for the consistent seedling emergence less affected by environmental variables and water availability in further research.

The GWAS population was assembled from global wheat collections, such as commercial varieties cultivars, inbred lines, and landraces to represent sufficient genetic diversity. There was low diversity in the first three PCs accounting for 17.6% of the total genetic variance. The closer genetic relationship between genotypes sourced from ICARDA and CIMMYT with those of Australian cultivars is likely a reflection of the historical and extensive germplasm exchange between the global breeding programs progressing toward a genetic bottleneck (Arruda et al., 2016; Francki et al., 2020). However, landraces sourced from dry origins, such as the middle-east, South West Asia, and North Africa were included to broaden the genetic base with potentially new phenotypic variation (Lopes et al., 2015; Liu et al., 2019). Landraces had a distinct grouping between genotypes from the breeding programs in Australia, ICARDA, and CIMMYT, indicating their value in broadening the genetic base for seedling emergence under limited water availability. Landrace entries, such as 013HAT10 and 010HAT10, with mean values > 85% for seedling emergence evaluated across all environments, and 066HAT10 and 087HAT10 with the greatest coleoptile and shoot length, respectively, provided credence to their importance in contributing alternative alleles for desirable phenotypes that are not necessarily represented in the current commercial breeding gene pool.

Genome-wide association study provided an initial analysis of genetic control of seedling emergence when the population was evaluated in the field and GH environments. The LD of the A and B-subgenomes was estimated to be higher than that for the D subgenome, which is typical of several GWAS studies of hexaploid wheat (Chao et al., 2010; Bajgain et al., 2015; Francki et al., 2020). Despite environmental interactions, GWAS identified 21 environment-specific QTL associated with seedling emergence when evaluated in the field or GH. It appears, therefore, that numerous genetic loci contribute to the phenotypic variation for seedling emergence, many of which are likely to be influenced by different environmental cues. Increasing evidence for copy number variants, presence/absence variants, insertion-deletion, and SNP events of gene sequences amongst modern and historical wheat cultivars defined by pan-genome sequencing (Montenegro et al., 2017; Walkowiak et al., 2020) indicate genomic structural variation at many loci may contribute to phenotypic plasticity. Therefore, it is conceivable that genomic structural variation amongst cultivars and landraces in the GWAS population may contribute to the alternative responses in seedling emergence, indicated by small or no correlation and lack of common QTL detected between most environments. In contrast, high phenotypic correlation but the lack of common QTL detected between field and GH experiments in 2019 indicated genetic variation within the population contributed to cumulative influence of different loci, such as those with possible small effects but below statistical power for detection in the GWAS analysis. The cumulative effect of environment-specific QTL of small and large effects and associated gene variants contributing to high phenotypic correlation was recently reported for fungal disease response in wheat (Joukhadar et al., 2020; Francki et al., 2021; Li et al., 2021).

We investigated the potential association of QTL with specific traits and genes of known function that could underpin the variation for seedling emergence. It was anticipated that direct analysis of QTL for coleoptile characteristics evaluated in controlled environments in this study would align to QTL for seedling emergence. However, only the QTL for coleoptile length on chromosome 1B, *QCL.daw.1B-1*, was in LD with *QSe.daw-1B-1* detected in May 2017 experiment. No further QTL for coleoptile characteristics were in LD with the remaining 20 QTL controlling seedling emergence in the field or GH environments. Therefore, it appears that environmental variation has a significant effect on coleoptile traits with few loci having a limited influence controlling the seedling emergence when evaluated in the field or the GH in 2017–2019. However, we cannot exclude the possibility that other loci for coleoptile characteristics are effective in field and GH environments. Therefore, we extended the analysis and incorporated previously reported QTL for coleoptile and shoot traits (Rebetzke et al., 2014; Li et al., 2017; Ma et al., 2020; Sidhu et al., 2020) by anchoring associated genetic markers and those in LD with seedling emergence detected from 2017–2019 experiments onto the high-quality wheat reference genome sequence (The International Wheat Genome Sequencing Consortium, 2014, 2018). Known QTL for coleoptile thickness, width, length, and shoot length (Rebetzke et al., 2014; Sidhu et al., 2020) were in LD with QTL controlling the seedling emergence on chromosomes 1B, 2B, and 4B, respectively, indicating that alternative loci controlling coleoptile characteristics can affect the seedling emergence in different environments. The nature of alternative loci is unclear but increasing evidence that genomic structural variants across genotypes (Gabur et al., 2019; Della Coletta et al., 2021) indicate that genomic differences within the GWAS population contribute to phenotypic variation for coleoptile and shoot traits associated with seedling emergence in different environments. Pangenome sequencing of selected individuals from the GWAS population would provide further clues on relationships between gene diversity amongst wheat genotypes and variation controlling seedling emergence under limited water availability in different environments.

The complex process of seedling emergence involves the coordinated response of physiological, biochemical, and molecular events associated with many traits, such as germination, dormancy, and vigor (Kucera et al., 2005; Weitbrecht et al., 2011; Rajjou et al., 2012). Therefore, we aligned QTL for seedling emergence and genetic markers associated with known loci and genes for specific traits onto the high-quality wheat reference genome sequence (The International Wheat Genome Sequencing Consortium, 2014, 2018). The analysis was restricted to the previously reported QTL studies for pre-harvest sprouting (Albrecht et al., 2015; Lin et al., 2016; Martinez et al., 2018; Zhu et al., 2019) germination percentage (Tarawneh et al., 2019) and ratio (Zuo et al., 2019), grain color (Lin et al., 2016), and seed longevity (Arif and Börner, 2020), where genetic markers could be unambiguously assigned to the reference genome sequence whereby 11 of the 21 QTL detected for seedling emergence were in LD with at least one QTL for each trait. Similarly, genes involved in hormone signaling for germination (Appleford et al., 2006; Yu et al., 2016; Izydorczyk et al., 2018), dormancy (Yu et al., 2020), and anthocynanin production in coleoptile development (Ahmed et al., 2009) were in LD with five QTL. Therefore, it appears that seedling emergence is controlled by a complex network of interrelated genes and traits regulated in different environments. The influence of restricted water availability and other environmental interactions on gene function for traits controlling seedling emergence is yet to be determined. Neither loci nor genes were in LD for the remaining five of the 21 QTL, so we cannot exclude that other QTL and genes for traits not identified in this study or previously reported playing an integral role in regulating the seedling emergence. Genome-wide sequencing of selected genotypes and gene-phenotype analysis would provide better opportunities to make inferences about the genes that cause variation and the role of interrelated traits giving rise to the differences in seedling emergence under limited water availability and the presence of alternative environmental cues.

## Conclusion

The ability of wheat seedlings to consistently emerge when rainfall is intermittent is the key to crop establishment under limited water without the need for deep sowing (>10 cm). Despite significant and high environment and genotype × environment interactions in six field and three GH experiments, high seedling emergence (>85%) was consistent for eight wheat genotypes across nine environments when sown at 4 cm depth with simulated rainfall events of 15–30 mm. Two landraces were included, one inbred line and five commercial lines representing genetically diverse germplasm that could be used as parental material in breeding. Therefore, not surprisingly, a total of 21 QTL were detected as environment-specific, indicating a complex interaction of genes and traits controlling seedling emergence likely to be regulated under different environmental cues. The underlying genes may regulate traits, such as coleoptile characteristics, pre-harvest sprouting and dormancy, germination, seed longevity, and anthocynanin production in coleoptile development. Genomic differences between individuals in the GWAS population may give rise to gene variation controlling pathways of interconnected traits in a multifaceted network of biological processes resulting in phenotypic variation for seedling emergence in different environments.

## Data Availability Statement

The raw data supporting the conclusions of this article will be made available by the authors, without undue reservation.

## Author Contributions

MGF acquired the research funding, contributed to the designing field and glasshouse experiments, coordinated the genomic, QTL and biometric analysis, interpreted the phenotyping and genotyping data, and wrote the manuscript. GSS implemented the field and glasshouse experiments, acquired the data, and contributed to the writing the manuscript. EW acquired and interpreted data for QTL and gene analysis and contributed to the writing the manuscript. GJR designed the experiments, acquired data, performed the biometric analysis, and interpreted the results for coleoptile characteristics, and contributed to the writing the manuscript. KTS designed the field and glasshouse experiments, performed the biometric analysis of data, and contributed to the writing the manuscript. RJF instigated the research idea for evaluation for wheat establishment under limited water availability, acquired the research funding, contributed to the experimental design, acquired and interpreted the data from field and glasshouse experiments and edited the manuscript. All authors read and approved the manuscript.

## Conflict of Interest

The authors declare that the research was conducted in the absence of any commercial or financial relationships that could be construed as a potential conflict of interest.

## Publisher’s Note

All claims expressed in this article are solely those of the authors and do not necessarily represent those of their affiliated organizations, or those of the publisher, the editors and the reviewers. Any product that may be evaluated in this article, or claim that may be made by its manufacturer, is not guaranteed or endorsed by the publisher.

## Footnotes

1
www.caigeproject.org.au

2
https://plantinformatics.io/

3
https://urgi.versailles.inra.fr/

## References

[B1] Abdi H. (2007). Bonferroni and Šidák corrections for multiple comparisons. *Encyc. Measure. Stat*. 3 103–107.

[B2] Addae P. C. Pearson C. J. (1992). Thermal requirements for germination and seedling growth of wheat. *Aust. J. Agric. Res.* 43 585–594. 10.1071/ar9920585

[B3] Ahmed N. Maekawa M. Noda K. (2009). Anthocyanin accumulation and expression pattern of anthocyanin biosynthesis genes in developing wheat coleoptiles. *Biol. Plant* 53 223–228. 10.1007/s10535-009-0043-x

[B4] Akinci C. Yildirim M. Bahar B. (2008). The effect of seed size on emergence and yield of durum wheat. *J. Food Agric. Env.* 6 234–237.

[B5] Alaux M. Rogers J. Letellier T. Flores R. Alfama F. Pommier C. (2018). Linking the international wheat genome sequencing consortium bread wheat reference genome sequence to wheat genomic and phenomic data. *Genome Biol.* 19:111. 10.1186/s13059-018-1491-4 30115101 PMC6097284

[B6] Albrecht T. Oberforster M. Kempf H. Ramgraber L. Schacht J. Kazman E. (2015). Genome-wide association mapping of pre-harvest sprouting resitance in a diversity panel of European winter wheats. *J. Appl. Genet.* 56 277–285. 10.1007/s13353-015-0286-5 25924791

[B7] Alomari D. Z. Eggert K. von Wirén N. Pillen K. Rőder M. S. (2017). Genome-wide association study of calcium accumulation in grains of European wheat cultivars. *Front. Plant Sci.* 8:1797. 10.3389/fpls.2017.01797 29163559 PMC5663994

[B8] Anzooman M. Christopher J. Mumford M. Dang Y. P. Menzies N. W. Kopittke P. M. (2018). Selection for rapid germination and emergence may improve wheat seedling establishment in the presence of soil surface crusts. *Plant Soil* 426 227–239.

[B9] Aoki N. Scofield G. N. Wang X.-D. Offler C. Patrick J. W. Furbank R. T. (2006). Pathway of sugar transport in germinating wheat seeds. *Plant Physiol.* 141 1255–1263. 10.1104/pp.106.082719 16766668 PMC1533920

[B10] Aoki N. Whitfeld P. Hoeren F. Scofield G. Newell K. Patrick J. (2002). Three sucrose transporter genes are expressed in the developing grain of hexaploid wheat. *Plant Mol. Biol.* 50 453–462. 10.1023/a:1019846832163 12369621

[B11] Appleford N. E. J. Evans D. J. Lenton J. R. Gaskin P. Croker S. J. Devos K. M. (2006). Function and transcript analysis of gibberellin-biosynthetic enzymes in wheat. *Planta* 223 568–582. 10.1007/s00425-005-0104-0 16160850

[B12] Arif M. A. R. Börner A. (2020). An SNP based GWAS analysis of seed longevity in wheat. *Cereal Res. Comm.* 48 149–156. 10.1007/s42976-020-00025-0

[B13] Arruda M. P. Brown P. Brown-Guedira G. Krill A. M. Thurber C. Merrill K. R. (2016). Genome-wide association mapping of fusarium head blight resistance in wheat using genotype-by-sequencing. *Plant Genome* 9:1 10.3835/plantgenome2015.04.0028 27898754

[B14] Bajgain P. Rouse M. N. Bulli P. Bhavani S. Gordon T. Wanyera R. (2015). Association mapping of North American spring wheat breeding germplasm reveals loci conferring resistance to Ug99 and other African stem rust races. *BMC Plant Biol.* 15:249. 10.1186/s12870-015-0628-9 26467989 PMC4606553

[B15] Belnap J. Prasse R. Harper K. T. (2001). “Influence of biological soil crusts on soil environments and vascular plants,” in *Biological Soil Crusts: Structure, Function, and Management. Ecological Studies (Analysis and Synthesis)*, Vol. 150 eds Belnap J. Lange O. L. (Berlin: Springer).

[B16] Bland J. M. Altman D. G. (1995). Multiple significance tests: the Bonferroni method. *BMJ* 310:170. 10.1136/bmj.310.6973.170 7833759 PMC2548561

[B17] Botwright T. L. Rebetzke G. J. Condon A. G. Richards R. A. (2005). Influence of the gibberellin-sensitive *Rht8* dwarfing gene on leaf epidermal cell dimensions and early vigour in wheat (*Triticum aestivum* L.). *Ann. Bot.* 95 631–639. 10.1093/aob/mci069 15655105 PMC4246859

[B18] Bradbury P. J. Zhang Z. Kroon D. E. T. Casstevens M. Ramdoss Y. Buckler E. S. (2007). TASSEL: software for association mapping of complex traits in diverse samples. *Bioinformatics* 23 2633–2635. 10.1093/bioinformatics/btm308 17586829

[B19] Buriro M. Oad F. C. Keerio M. I. Tunio S. Gandahu A. W. Ul Hassan S. W. (2011). Wheat seed germination under the influence of temperature regimes. *Sarhad J. Agric.* 27 539–543.

[B20] Cai W. Cowan T. (2013). Southeast Australia autumn rainfall reduction: a climate-change-induced poleward shift of ocean-atmosphere circulation. *J. Climate* 26 189–205.

[B21] Carpita N. C. (1996). Structure and biogenesis of the cell walls of grasses. *Ann. Rev. Plant Phyiol. Mol. Biol.* 47 445–476. 10.1146/annurev.arplant.47.1.445 15012297

[B22] Carpita N. C. Defernez M. Findlay K. Wells B. Shoue D. A. Catchpole G. (2001). Cell wall architecture of the elongating maize coleoptile. *Plant Physiol.* 127 551–565. 11598229 PMC125090

[B23] Chao S. Dubcovsky J. Dvorak J. Luo M.-C. Baenziger S. P. Matnyazov R. (2010). Population and genome-specific patterns of linkage disequilibrium and SNP variation in spring and winter wheat (*Triticum aestivum* L.). *BMC Genom*. 11:727. 10.1186/1471-2164-11-727 21190581 PMC3020227

[B24] Coombes N. E. (2002). *The Reactive Tabu Search for Efficient Correlated Experimental Designs.* PhD Thesis. Liverpool: Liverpool John Moores University.

[B25] Della Coletta R. Qiu Y. Ou S. Hufford M. B. Hirsch C. N. (2021). How the pan-genome is changing crop genomics and imporvement. *Genome Biol.* 22:3. 10.1186/s13059-020-02224-8 33397434 PMC7780660

[B26] Forcella F. Benech Arnold R. L. Sanchez R. Ghersa C. M. (2000). Modelling seedling emergence. *Field Crops Res.* 67 123–139.

[B27] Francki M. G. Walker E. McMullan C. J. Morris W. G. (2020). Multi-location evaluation of global wheat lines reveal mutliple QTL for adult plant resistance to Septoria nodorum blotch (SNB) detected in specific environments and in repsosne to different isolates. *Front. Plant Sci.* 11:771. 10.3389/fpls.2020.00771 32655592 PMC7325896

[B28] Francki M. G. Walker E. McMullan C. J. Morris W. G. (2021). Evaluation of Septoria nodorum blotch (SNB) resistance in glumes of wheat (*Triticum aestivum* L.) and the genetic relationship with foliar disease response. *Front. Genet.* 12:681768. 10.3389/fgene.2021.681768 34267781 PMC8276050

[B29] Gabriel S. Schaffner S. F. Nguyen H. Moore J. M. Roy J. Blumenstiel B. (2002). The structure of haplotype blocks in the human genome. *Science* 296 2225–2229. 10.1126/science.1069424 12029063

[B30] Gabur I. Chawla H. S. Snowdon R. J. Parkin I. A. P. (2019). Connecting genome structural variation with complex traits in crop plants. *Theor. Appl. Genet.* 132 733–750. 10.1007/s00122-018-3233-0 30448864

[B31] Gao D.-Y. Xu Z.-S. He Y. Sun Y.-W. Ma Y.-Z. Xia L.-Q. (2014). Functional analyses of an E3 ligase gene *AIP2* from wheat in *Arabidopsis* revealed its role in seed germination and pre-harvest sprouting. *J. Integr. Plant Biol.* 56 480–491. 10.1111/jipb.12135 24279988

[B32] Gao X. Hu C. H. Li H. Z. Yao Y. J. Meng M. Dong J. (2013). Factors affecting pre-harvest sprouting resistance in wheat (*Triticum aestivum* L.): a review. *J. Anim. Plant Sci.* 23 556–565.

[B33] Gooding S. Botwright T. L. Fox P. N. Wade L. J. (2006). Emergence, stand establishment and vigour of deep-sown Australian and CIMMYT wheat. *Aust. J. Exp. Agric.* 46 1167–1175. 10.1071/ea05200

[B34] Himi E. Maekawa M. Noda K. (2011). Differential expression of three flavanone 3-hydroxylase genes in grains and coleoptiles of wheat. *Int. J. Plant Genom.* 2011:369460. 10.1155/2011/369460 21977025 PMC3185259

[B35] Himi E. Nisar A. Noda K. (2005). Colour genes (R and Rc) for grain and coleoptile upregulate flavonoid biosynthesis genes in wheat. *Genome* 48 747–754. 10.1139/g05-026 16094442

[B36] Hochman Z. Gobbett D. L. Horan H. (2017). Climate trends account for stalled wheat yields in Australia since 1990. *Glob. Change Biol.* 23 2071–2081. 10.1111/gcb.13604 28117534

[B37] Hunt J. R. Lilley J. M. Trevaskis B. Flohr B. M. Peake A. Fletcher A. (2019). Early sowing systems can boost Australian wheat yields despite recent climate change. *Nat. Clim. Change* 9 244–247.

[B38] Izydorczyk C. Nguyen T.-N. Jo S. Son S. Tuan P. Ayele B. Y. (2018). Spatiotemporal modulation of abscisic acid and gibberellin metabolism and signalling mediates the effects of suboptimal and supraoptimal temperatures on seed germination in wheat (*Triticum aestivum* L.). *Plant Cell Environ.* 41 1022–1037. 10.1111/pce.12949 28349595

[B39] Joukhadar R. Hollaway G. Shi F. Kant S. Forrest K. Wong D. (2020). Genome-wide association reveals a complex architecture for rust resistance in 2300 worldwide bread wheat accessions screened under various Australian conditions. *Theor. Appl. Genet*. 133 2695–2712. 10.1007/s00122-020-03626-9 32504212

[B40] Keeble-Gagnère G. Isdale D. Suchecki R. Kruger A. Lomas K. Carroll D. (2019). Integrating past, present and future wheat research with Pretzel. *bioRXiv [Preprint]* 10.1101/517953

[B41] Keyes G. J. Paolillo D. J. Sorrells M. E. (1989). The effects of dwarfing genes Rht1 and Rht2 on cellular dimensions and rate of leaf elongation in wheat. *Ann. Bot.* 64 683–690. 10.1093/oxfordjournals.aob.a087894

[B42] Khah E. M. Ellis R. H. Roberts E. H. (1986). Effects of laboratory germination, soil temperature and moisture content on the emergence of spring wheat. *J. Agric. Sci. Camb.* 107 431–438. 10.1017/s0021859600087232

[B43] Kim Y. J. Kim J. Y. Yoon J. S. Kim D. Y. Hong M. J. Seo Y. W. (2016). Characterization of 4 *TaGAST* genes during spike development and seed germination and their response to exogenous phytohormones in common wheat. *Mol. Biol. Rep.* 43 1435–1449. 10.1007/s11033-016-4077-9 27649990

[B44] Kucera B. Cohn M. A. Leubner-Metzger G. (2005). Plant hormone interactions during seed dormancy release and germination. *Seed Sci. Res.* 4 281–307. 10.1079/ssr2005218

[B45] Lei L. Zhu X. Wang S. Zhu M. Carver B. F. Yan L. (2013). *TaMFT-A1* is associated with seed germination sensitive to temperature in winter wheat. *PLoS One* 8:e73330. 10.1371/journal.pone.0073330 24069187 PMC3772017

[B46] Li D. Walker E. Francki M. G. (2021). Genes associated with foliar resistance to Septoria nodorum blotch of hexaploid wheat (*Triticum aestivum* L.). *Int. J. Mol. Sci.* 22:5580. 10.3390/ijms22115580 34070394 PMC8197541

[B47] Li G. Bai G. Varver B. F. Elliott N. C. Bennett R. S. Wu Y. (2017). Genome-wide association study reveals genetic architecture of coleoptile length in wheat. *Theor. Appl. Genet.* 130 391–401. 10.1007/s00122-016-2820-1 27844116

[B48] Lin M. Zhang D. Liu S. Zhang G. Yu J. Fritz A. K. (2016). Genome-wide association analysis on pre-harvest sprouting resistance and grain colour in U.S. winter wheat. *BMC Genom.* 17:794. 10.1186/s12864-016-3148-6 27729004 PMC5059910

[B49] Liu A. Gao F. Kanno Y. Jordan M. C. Kamiya Y. Seo M. (2013). Regulation of wheat seed dormancy by after-ripening is mediated by specific transcriptional switches that induces changes in seed hormone metabolism and signaling. *PLoS One* 8:e56570. 10.1371/journal.pone.0056570 23437172 PMC3577873

[B50] Liu J. Rasheed A. He Z. Imtiaz M. Arif A. Mahmood T. (2019). Genome-wide variation patterns between landraces and cultivars uncover divergent selection during modern wheat breeding. *Theor. Appl. Genet.* 132 2509–2523. 10.1007/s00122-019-03367-4 31139853

[B51] Lopes M. S. Dreisigacker S. Peña R. J. Sukumaran S. Reynolds M. P. (2015). Genetic characterization of the wheat association mapping initiative (WAMI) panel for dissection of complex traits in spring wheat. *Theor.Appl. Genet.* 128 453–464. 10.1007/s00122-014-2444-2 25540818

[B52] López-Castaneda C. Richards R. A. Farquhar G. D. Williamson R. E. (1996). Seed and seedling characteristics contributing to variation in early vigour among temperate cereals. *Crop Sci.* 36 1257–1266. 10.2135/cropsci1996.0011183x003600050031x 34798789

[B53] Ma J. Lin Y. Tang S. Duan S. Wang Q. Wu F. (2020). A genome-wide association study of coleoptile length in different Chinese wheat landraces. *Front. Plant Sci.* 11:677. 10.3389/fpls.2020.00677 32582239 PMC7287122

[B54] Maccaferri M. Zhang J. Bulli P. Abate Z. Chao S. Canto D. (2015). A genome-wide association study of resistance to stripe rust (*Puccinia striiformis* f. sp. *tritici*) in a worldwide collection of hexaploid spring wheat. *Genes Genom. Genet.* 5 449–465. 10.1534/g3.114.014563 25609748 PMC4349098

[B55] Maccaferri M. El-Feki W. Nazemi G. Salvi S. Canè M. A. Colalongo M. C. (2016). Prioritizing quantitative trait loci for root system architecture in tetraploid wheat. *J. Exp. Bot.* 67 1161–1178. 10.1093/jxb/erw039 26880749 PMC4753857

[B56] Marroni S. Pinosio G. Zaina F. Fogolari N. Felice F. Cattonaro F. (2011). Nucleotide diversity and linkage disequilibrium in *Populus nigra* cinnamyl alcohol dehydrogenase (*CAD4*) gene. *Tree Genet. Genomes* 7 1011–1023. 10.1007/s11295-011-0391-5

[B57] Martinez S. A. Godoy J. Huang M. Zhang Z. Carter A. H. Garland Campbell K. A. (2018). Genome-wide association mapping for tolerance to preharvest sprouting and low falling numbers in wheat. *Front. Plant Sci.* 9:141. 10.3389/fpls.2018.00141 29491876 PMC5817628

[B58] Mohan A. Schillinger W. F. Gill K. S. (2013). Wheat seedling emergence from deep planting depths and its relationship with coleoptile length. *PLoS One* 8:e73314. 10.1371/journal.pone.0073314 24019916 PMC3760894

[B59] Montenegro J. D. Golicz A. A. Bayer P. E. Hurgobin B. Lee H. Chan C.-K. K. (2017). The pangenome of hexaploidy bread wheat. *Plant J*. 90 1007–1013. 10.1111/tpj.13515 28231383

[B60] Montieth J. L. (1965). Evaporation and environment. *Symp. Soc. Exp. Biol.* 19 205–234. 5321565

[B61] Muqaddasi Q. H. Zhao Y. Rodemann B. Plieske J. Ganal M. W. Röder M. S. (2019). Genome-wide association mapping and prediction of adult stage Septoria tritici blotch infection in European winter wheat via high-density marker arrays. *Plant Genome* 12:180029. 10.3835/plantgenome2018.05.0029 30951099 PMC12809923

[B62] Nakamura S. Abe F. Kawahigashi H. Nakazono K. Tagiri A. Matsumoto T. (2011). A wheat homolog of MOTHER OF FT AND TFL1 acts in the regulation of germination. *Plant Cell* 23 3215–3229. 10.1105/tpc.111.088492 21896881 PMC3203438

[B63] Nakamura S. Komatsuda T. Miura H. (2007). Mapping and diploid wheat homologues of *Arabidopsis* seed ABA signaling genes and QTLs for seed dormancy. *Theor. Appl. Genet.* 114 1129–1139. 10.1007/s00122-007-0502-8 17387417

[B64] Nasr H. M. Selles F. (1995). Seedling emergence as influenced by aggregate size, bulk density, and penetration resistance of the seed bed. *Soil Till. Res.* 34 61–76. 10.1016/0167-1987(94)00451-j

[B65] Pearce S. Huttly A. K. Prosser I. M. Li Y. Vaughan S. P. Gallova B. (2015). Heterologous expression and transcript analysis of gibberellin biosynthetic genes of grasses reveals novel functionality in the *GA3ox* family. *BMC Plant Biol.* 15:130. 10.1186/s12870-015-0520-7 26044828 PMC4455330

[B66] Penfield S. MacGregor D. R. (2017). Effects of environmental variation during seed production on seed dormancy and germination. *J. Exp. Bot.* 68 819–825. 10.1093/jxb/erw436 27940467

[B67] Photiades I. Hadjichristodoulou A. (1984). Sowing date, sowing depth, seed rate and row spacing of wheat and barley under dryland conditions. *Field Crops Res.* 9 151–162. 10.1016/0378-4290(84)90021-2

[B68] Purcell S. Neale B. Todd-Brown K. Thomas L. Ferreira M. A. R. Bender D. (2007). PLINK: a toolset for whole-genome association and population-based linkage analysis. *Am. J. Human Genet.* 81 559–579. 10.1086/519795 17701901 PMC1950838

[B69] R Core Team (2018). *R: A Language and Environment for Statistical Computing.* Vienna: R Foundation for Statistical Computing.

[B70] Rajjou L. ZDuval M. Gallardo K. Catusse J. Bally J. Job C. (2012). Seed germination and vigour. *Ann. Rev. Plant Biol.* 63 507–533. 22136565 10.1146/annurev-arplant-042811-105550

[B71] Rebetzke G. J. Richards R. A. (2000). Gibberellic acid-sensitive dwarfing genes reduce plant height to increase kernel number and grain yield of wheat. *Aus. J. Agric. Res.* 51 235–246. 10.1071/ar99043

[B72] Rebetzke G. J. Ellis M. H. Bonnett D. G. Richards R. A. (2007). Molecular mapping of genes for coleoptile growth in bread wheat (*Triticum aestivum* L). *Theor. Appl. Genet.* 114 1173–1183. 10.1007/s00122-007-0509-1 17294164

[B73] Rebetzke G. J. Richards R. A. Fischer V. M. Mickelson B. J. (1999). Breeding long coleoptile, reduced height wheats. *Euphytica* 106 159–168.

[B74] Rebetzke G. J. Richards R. A. Sirault X. R. R. Morrison A. D. (2004). Genetic analysis of coleoptile length and diameter of wheat. *Aus. J. Agric. Res.* 55 733–743. 10.1071/ar04037

[B75] Rebetzke G. J. Verbyla A. Verbyla K. Morell M. Cavanagh C. (2014). Use of a large multiparent wheat mapping population for genomic dissection of coleoptile and seedling growth. *Plant Biotech J.* 12 219–230. 10.1111/pbi.12130 24151921

[B76] Rebetzke G. J. Zheng B. Chapman S. C. (2016). Do wheat breeders have suitable genetic variation to overcome short coleoptiles and poor establishment in the warmer soils of future climates? *Funct. Plant Biol.* 43 961–972. 10.1071/FP15362 32480519

[B77] Russell J. J. (2005). *Major Eastern Wheatbelt Soils to Characterise Soil Moisture Availability.* Perth: Department of Agriculture and Food. Report 187.

[B78] Saeidi G. (2012). Genetic variation and heritability for germination, seed vigour and field emergence in brown and yellow-seeded genotpyes of flax. *Int. J. Plant Prod.* 2 15–22.

[B79] Scanlon T. T. Doncon G. (2020). Rain, rain, gone away: decreased growing-season rainfall for the dryland cropping region of the south-west of Western Australia. *Crop Past. Sci.* 71 128–133. 10.1071/cp19294

[B80] Schillinger W. F. Donaldson E. Allan R. E. Jones S. S. (1998). Winter wheat seedling emergence from deep sowing depths. *Agron. J.* 90 582–586. 10.2134/agronj1998.00021962009000050002x

[B81] Shackley B. J. Anderson W. K. (1995). Responses of wheat cultivars to time of sowing in the southern wheatbelt of Western Australia. *Aus. J. Exp. Agric.* 35 579–587. 10.1071/ea9950579

[B82] Shin D. H. Choi M.-G. Kang C.-S. Park C.-S. Choi S.-B. Parl Y.-I. (2016). A wheat R2R3-MYB protein PURPLE PLANT1 (TaPL1) functions as a positive regulator of anthocyanin biosynthesis. *Biochem. Biophys. Res. Comm.* 469 686–691. 10.1016/j.bbrc.2015.12.001 26692488

[B83] Shoeva O. Y. Gordeeva E. I. Khlestkina E. K. (2014). The regulation of anthocyanin synthesis in the wheat pericarp. *Molecules* 19 20266–20279. 10.3390/molecules191220266 25486242 PMC6271175

[B84] Sidhu J. S. Singh D. Gill H. S. Brar N. K. Qiu Y. Halder J. (2020). Genome-wide association study uncovers novel genomic regions associated with coleoptile length in hard winter wheat. *Front. Genet.* 10:1345. 10.3389/fgene.2019.01345 32117410 PMC7025573

[B85] Singh N. T. Dhaliwal G. S. (1972). Effects of soil temperature on seedling emergence in different crops. *Plant Soil* 37 441–444. 10.1007/bf02139989

[B86] Singla B. Chugh A. Khurana J. P. Khurana P. (2006). An early auxin-responsive *Aux/IAA* gene from wheat (*Triticum aestivum*) is induced by epibrassinolide and differentially regulated by light and calcium. *J. Exp. Bot.* 57 4059–4070. 10.1093/jxb/erl182 17077182

[B87] Smith A. Lim P. Cullis B. (2006). The design and analysis of multi-phase plant breeding experiments. *J. Agric. Sci.* 144 393–409.

[B88] Son S. Chitnis V. R. Liu A. Gao F. Nguyen T.-N. Ayele B. T. (2016). Abscisic acid metabolic genes of wheat (*Triticum aestivum* L.): identification and insights into their functionality in seed dormancy and dehydration tolerance. *Plant* 244 429–447. 10.1007/s00425-016-2518-2 27091738

[B89] Spielmeyer W. Hyles J. Jaoquim P. Azanza F. Bonnett D. G. Ellis M. E. (2007). A QTL on chromosome 6A in bread wheat (*Triticum aestivum*) is associated with longer coleoptiles, greater seedling vigour and final plant height. *Theor. Appl. Genet.* 115 59–66. 10.1007/s00122-007-0540-2 17429602

[B90] Stephens D. J. Lyons T. J. (1998). Variability and trends in sowing dates across the Australian wheatbelt. *Aust. J. Agric. Res.* 49 1111–1118. 10.1093/jxb/erv163 25922479 PMC4463805

[B91] Strygina K. Khlestkina E. K. (2019). Structural and functional divergence of the Mpc1 genes in wheat and barley. *BMC Evol Biol*. 19:89–99. 10.1186/s12862-019-1378-3 30813913 PMC6391766

[B92] Tarawneh R. A. Szira F. Monostori I. Behrens A. Alqudah A. M. Thumm S. (2019). Genetic analysis of drought response of wheat following either chemical dessication or the use of a rain-out shelter. *J. Appl. Genet.* 60 137–146. 10.1007/s13353-019-00494-y 30949857

[B93] The International Wheat Genome Sequencing Consortium (2014). A chromosome-based draft sequence of the hexaploid bread wheat (*Triticum aestivum*) genome. *Science* 345 6194. 10.1126/science.1251788 25035500

[B94] The International Wheat Genome Sequencing Consortium (2018). Shifting the limits of wheat research and breeding using a fully annotated reference genome. *Science* 361 eaar7191. 10.1126/science.aar7191 30115783

[B95] Torada A. Koike M. Ogawa T. Takenouchi Y. Tadamura K. Wu J. (2016). A causal gene for seed dormancy on wheat chromosome 4A encodes a MAP kinases kinase. *Curr. Biol.* 26 782–787. 10.1016/j.cub.2016.01.063 26948878

[B96] Turner S. (2017). *qqman: Q-Q and Manhattan Plots for GWAS Data. R Package Version 0.1.4.* Available online at: https://CRAN.R-project.org/package=qqman

[B97] Vetch J. M. Stougaard R. N. Martin J. M. Giroux M. J. (2019). Review: revealing the genetic mechanisms of pre-harvest sprouting in hexaploidy wheat (*Triticum aestivum* L.). *Plant Sci.* 281 180–185. 10.1016/j.plantsci.2019.01.004 30824050

[B98] VSN International (2020). *Genstat for Windows*, 21st Edn. Hemel Hempstead: VSN International.

[B99] Walkowiak S. Gao L. Monat C. Haberer G. Kassa M. T. Brinton J. (2020). Multiple wheat genomes reveal global variation in modern breeding. *Nature* 588:277. 10.1038/s41586-020-2961-x 33239791 PMC7759465

[B100] Wang S. Wong D. Forrest K. Allen A. Chao S. Huang B. E. (2014). Characterization of polyploid wheat genomic diversity using a high-density 90 000 single nucleotide polymorphism array. *Plant Biotech. J*. 12 787–796. 10.1111/pbi.12183 24646323 PMC4265271

[B101] Weitbrecht K. Műller K. Leubner-Metzger G. (2011). First off the mark: early seed germination. *J. Exp. Bot.* 62 3289–3309. 10.1093/jxb/err030 21430292

[B102] Wuest S. B. Albrecht S. L. Skirvin K. W. (1999). Vapour transport vs. seed-soil contact in wheat germination. *Agron. J.* 91 783–787.

[B103] Xing S.-C. Li F. Guo Q.-F. Liu D.-R. Zhao W.-X. Wang W. (2009). The involvement of an expansin gene *TaEXPB23* from wheat in regulating plant cell growth. *Biol. Plant* 53 429–434.

[B104] Yang Y. Zhao X. L. Xia L. Q. Chen X. M. Xia X. C. Yu Z. (2007). Development and validation of a *Viviparous-1* STS marker for pre-harvest sprouting tolerance in Chinese wheats. *Theor. Appl. Genet*. 115 971–980. 17712543 10.1007/s00122-007-0624-z

[B105] Yu X. Han J. Li L. Zhang Q. Yang G. He G. (2020). Wheat PP2C-a10 regulates seed germination and drought tolerance in transgenic *Arabidiopsis*. *Plant Cell Rep.* 39 635–651. 32065246 10.1007/s00299-020-02520-4 PMC7165162

[B106] Yu Y. Guo G. Lv D. Hu Y. Li J. Li X. (2014). Transcriptome analysis during seed germination of elite Chinese bread wheat cultivar Jimai 20. *BMC Plant Biol.* 14:20. 10.1186/1471-2229-14-20 24410729 PMC3923396

[B107] Yu Y. Zhen S. Wang S. Wang Y. Cao H. Zhang Y. (2016). Comparative transcriptome analysis of wheat embryo and endosperm responses to ABA and H_2_O_2_ during seed germination. *BMC Genom.* 17:97. 10.1186/s12864-016-2416-9 26846093 PMC4743158

[B108] Zhang Y. Miao X. Xia X. He Z. (2014). Cloning of seed dormancy genes (*TaSdr*) associated with tolerance to pre-harvest sprouting in common wheat and development of a functional marker. *Theor. Appl. Genet.* 127 855–866. 24452439 10.1007/s00122-014-2262-6

[B109] Zhu Y. Wang S. Wei W. Xie H. Liu K. Zhang C. (2019). Genome-wide assoication of pre-harvets sprouting tolerance using a 90K SNP array in common wheat (*Triticum aestivum* L). *Theor. Appl. Genet*. 132 2947–2963. 31324930 10.1007/s00122-019-03398-x

[B110] Zuo J. Lin C. Cao H. Chen F. Liu Y. Liu J. (2019). Genome-wide association study and quantitative trait loci mapping of seed dormancy in common wheat (*Triticum aestivum* L). *Planta* 250 187–198. 30972483 10.1007/s00425-019-03164-9

