# Supplementary material for: Phenotypic Evaluation and Genetic Analysis of Seedling Emergence in a Global Collection of Wheat Genotypes (Triticum aestivum L.) Under Limited Water Availability
Source: Front Plant Sci. 2021 Dec 24;12:796176. doi: 10.3389/fpls.2021.796176 (PMC8739788; doi:10.3389/fpls.2021.796176)
Supplement: Supplementary file 1 [file Table_1.DOCX]

| *Line #*  **Table S1.** List of wheat genotypes from Australia, CIMMYT, ICARDA including their pedigrees and global landraces used in phenotypic evaluation for field and glasshouse and genetic control GWAS analysis for seedling emergence in 2017-2019. | Entry | Origin | Pedigree |
| --- | --- | --- | --- |
| *1* | 001HAT10 | AFGHANISTAN | LANDRACE |
| *2* | 003HAT10 | PAKISTAN | LANDRACE |
| *3* | 004HAT10 | CHINA | LANDRACE |
| *4* | 005HAT10 | TAJIKISTAN | LANDRACE |
| *5* | 010HAT10 | PAKISTAN | LANDRACE |
| *6* | 012HAT10 | PAKISTAN | LANDRACE |
| *7* | 013HAT10 | PAKISTAN | LANDRACE |
| *8* | 016HAT10 | PAKISTAN | LANDRACE |
| *9* | 020HAT10 | KAZAKHSTAN | LANDRACE |
| *10* | 023HAT10 | TURKMENISTAN | LANDRACE |
| *11* | 024HAT10 | TAJIKISTAN | LANDRACE |
| *12* | 025HAT10 | KAZAKHSTAN | LANDRACE |
| *13* | 026HAT10 | CHINA | LANDRACE |
| *14* | 027HAT10 | CHINA | LANDRACE |
| *15* | 028HAT10 | INDIA | LANDRACE |
| *16* | 029HAT10 | INDIA | LANDRACE |
| *17* | 031HAT10 | SUDAN | LANDRACE |
| *18* | 032HAT10 | SUDAN | LANDRACE |
| *19* | 033HAT10 | SUDAN | LANDRACE |
| *20* | 035HAT10 | INDIA | LANDRACE |
| *21* | 036HAT10 | PAKISTAN | LANDRACE |
| *22* | 040HAT10 | PAKISTAN | LANDRACE |
| *23* | 041HAT10 | PAKISTAN | LANDRACE |
| *24* | 044HAT10 | PAKISTAN | LANDRACE |
| *25* | 048HAT10 | PAKISTAN | LANDRACE |
| *26* | 054HAT10 | PAKISTAN | LANDRACE |
| *27* | 059HAT10 | PAKISTAN | LANDRACE |
| *28* | 062HAT10 | PAKISTAN | LANDRACE |
| *29* | 066HAT10 | PAKISTAN | LANDRACE |
| *30* | 069HAT10 | PAKISTAN | LANDRACE |
| *31* | 070HAT10 | PAKISTAN | LANDRACE |
| *32* | 076HAT10 | PAKISTAN | LANDRACE |
| *33* | 080HAT10 | PAKISTAN | LANDRACE |
| *34* | 085HAT10 | PAKISTAN | LANDRACE |
| *35* | 087HAT10 | PAKISTAN | LANDRACE |
| *36* | 090HAT10 | PAKISTAN | LANDRACE |
| *37* | 093HAT10 | PAKISTAN | LANDRACE |
| *38* | 096HAT10 | PAKISTAN | LANDRACE |
| *39* | 097HAT10 | PAKISTAN | LANDRACE |
| *40* | 100HAT10 | PAKISTAN | LANDRACE |
| *41* | 107HAT10 | PAKISTAN | LANDRACE |
| *42* | 109HAT10 | PAKISTAN | LANDRACE |
| *43* | 110HAT10 | PAKISTAN | LANDRACE |
| *44* | 114HAT10 | PAKISTAN | LANDRACE |
| *45* | 116HAT10 | PAKISTAN | LANDRACE |
| *46* | 118HAT10 | PAKISTAN | LANDRACE |
| *47* | 124HAT10 | TURKEY | LANDRACE |
| *48* | 127HAT10 | PAKISTAN | LANDRACE |
| *49* | 131HAT10 | PAKISTAN | LANDRACE |
| *50* | 135HAT10 | JORDAN | LANDRACE |
| *51* | 138HAT10 | ALGERIA | LANDRACE |
| *52* | 139HAT10 | ALGERIA | LANDRACE |
| *53* | 142HAT10 | IRAN | LANDRACE |
| *54* | 146HAT10 | IRAQ | LANDRACE |
| *55* | 147HAT10 | IRAN | LANDRACE |
| *56* | 151HAT10 | IRAN | LANDRACE |
| *57* | 153HAT10 | IRAN | LANDRACE |
| *58* | 154HAT10 | IRAN | LANDRACE |
| *59* | 162HAT10 | IRAN | LANDRACE |
| *60* | 163HAT10 | IRAN | LANDRACE |
| *61* | 166HAT10 | IRAN | LANDRACE |
| *62* | 167HAT10 | IRAN | LANDRACE |
| *63* | 168HAT10 | IRAN | LANDRACE |
| *64* | 170HAT10 | TAJIKISTAN | LANDRACE |
| *65* | 3:ZIZ12 | ICARDA | TEVEE-3/SHUHA-20//SERI 82/SHUHA'S' |
| *66* | 4:ZIZ13 | ICARDA | TRACHA'S'//CMH76-252/PVN'S' |
| *67* | 5:ZIZ13 | ICARDA | CNDO/R143//ENTE/MEXI/3/AEGILOPSSQUARROSA(TAUS)/4/WEAVER/5/MYNA/VUL |
| *68* | 7:ZIZ13 | ICARDA | ZEMAMRA-5/SOMAMA-3 |
| *69* | 8:ZIZ13 | ICARDA | MUNIA//CHEN/ALTAR 84/3/CHEN/AEGILOPS SQUARROSA (TAUS)//BCN/4/MARCHOUCH-8 |
| *70* | 13:ZIZ13 | ICARDA | PAVON 76/HAMAM-4/4/YACO/PBW65/3/KAUZ*2/TRAP//KAUZ |
| *71* | 16:ZIZ13 | ICARDA | ZEMAMRA-8/3/SHA3/SERI//SHA4/LIRA/4/QAFZAH-21 |
| *72* | 20:ZIZ12 | ICARDA | SERI 82/SHUHA'S'//CM84655-02AP-300AP-300L-3AP-300L-3AP-0L-0AP |
| *73* | 21:ZIZ13 | ICARDA | ZEMAMRA-5/ZEMAMRA-5 |
| *74* | 23:ZIZ13 | ICARDA | TINAMOU-2//TEVEE-1/SHUHA-6 |
| *75* | 31:ZIZ13 | ICARDA | KARAWAN-1/TALLO 3//JADIDA-2 |
| *76* | 32:ZIZ12 | ICARDA | FERROUG-2/POTAM*2KS811261-8//ZEMAMRA-8 |
| *77* | 33:ZIZ12 | ICARDA | MON'S'/ALD'S'//ALDAN'S'/IAS58/3/SAFI-1/4/ZEMAMRA-1 |
| *78* | 33:ZIZ13 | ICARDA | SOUS-1/ARREHANE |
| *79* | 36:ZIZ13 | ICARDA | TEVEE-1/SHUHA-6//MASSIRA |
| *80* | 41:ZIZ13 | ICARDA | MASSIRA/SAFI-1 |
| *81* | 43:ZIZ13 | ICARDA | TEVEE-1/STAR'S'/3/ACHTAR*3//KANZ/KS85-8-4 |
| *82* | 45:ZIZ12 | ICARDA | HUBARA-8///MON'S'/ALD'S'//BOW'S' |
| *83* | 47:ZIZ13 | ICARDA | ACHTAR//ATTILA-1/NS732/HER |
| *84* | 48:ZIZ12 | ICARDA | QAFZAH-33/ICW84.0074-09AP-300L-1AP-300L-8AP-0L-0AP |
| *85* | 50:ZIZ13 | ICARDA | QAFZAH-21/OUEDZEM-1 |
| *86* | 51:ZIZ12 | ICARDA | SERI 82/SHUHA'S'//CM85295-0101TOPY-2M-0Y-0M-3Y-0M-0AP |
| *87* | 52:ZIZ12 | ICARDA | ICW91.0145-4AP-0TS-2AP-0L-0AP/4/NAI60/HN7//SX/3/JUN'S' |
| *88* | 53:ZIZ12 | ICARDA | ICW91.0145-4AP-0TS-2AP-0L-0AP/QAFZAH-33 |
| *89* | 53:ZIZ13 | ICARDA | MEXIPAK/FLORKWA-2 |
| *90* | 55:ZIZ13 | ICARDA | CHAM-4/SHUHA'S'/6/2*SAKER/5/RBS/ANZA/3/KVZ/HYS//YMH/TOB/4/BOW'S' |
| *91* | 56:ZIZ13 | ICARDA | SHUHA-7//SERI 82/SHUHA'S' |
| *92* | 61:ZIZ12 | ICARDA | NS732/HER//SAADA///SAADA |
| *93* | 72:ZIZ12 | ICARDA | ACHTAR/IG 132176 |
| *94* | 75:ZIZ13 | ICARDA | HUBARA-3*2/SHUHA-4 |
| *95* | 83:ZIZ12 | ICARDA | ESWYT99#18/ARRIHANE |
| *96* | 90:ZIZ13 | ICARDA | HD2281/PVN/3/KAUZ*2/TRAP//KAUZ/4/AALAAL-1 |
| *97* | 96:ZIZ13 | ICARDA | FLORKWA2/6/SAKER'S'/5/RBS/ANZA/3/KVZ/HYS//YMH/TOB/4/BOW'S'/7/DAJAJ-6 |
| *98* | 98:ZIZ13 | ICARDA | HIDDAB/ATTILA-7 |
| *99* | 104:ZIZ13 | ICARDA | HIDDAB/FLORKWA-2 |
| *100* | 109:ZIZ13 | ICARDA | QAFZAH-20/QIMMA-5//FERROUG-3 |
| *101* | 111:ZIZ13 | ICARDA | ATENA-1/GAMDOW-3/3/MON'S'/ALD'S'//ALDAN'S'/IAS58 |
| *102* | 112:ZIZ13 | ICARDA | CHAM-6/PERW//MILAN/PASTOR/3/CHAM-6/PERW |
| *103* | 115:ZIZ13 | ICARDA | HUBARA-8/3/MUNIA/ALTAR 84//MILAN/4/ANGI-2 |
| *104* | 116:ZIZ12 | ICARDA | INQALAB 91/CMSW94WM00188S-0300M-0100Y-0100M-13Y-4M-0Y-0AP |
| *105* | 123:ZIZ13 | ICARDA | QAFZAH-23/SOMAMA-3//GOUMRIA-3 |
| *106* | 124:ZIZ12 | ICARDA | CMT/ALD//ZARZOUR/5/AU//KAL/BB/3/BON/4/HPO |
| *107* | 127:ZIZ13 | ICARDA | TAZA-2/LAKTA-5//HAAMA-11 |
| *108* | 130:ZIZ13 | ICARDA | HUBARA-5/ANGI-1 |
| *109* | 137:ZIZ13 | ICARDA | ATTILA-7/SOMAMA-3 |
| *110* | 141:ZIZ13 | ICARDA | BJY/COC//PRL/BOW/3/BLOYKA-1 |
| *111* | 142:ZIZ13 | ICARDA | KAUZ'S'/BOCRO-3//ANGI-2 |
| *112* | 149:ZIZ13 | ICARDA | HUBARA-5/3/SHA3/SERI//SHA4/LIRA/4/QAFZAH-21 |
| *113* | 151:ZIZ13 | ICARDA | HUBARA-1/3/MUNIA/CHTO//MILAN/4/GOUMRIA-8 |
| *114* | 152:ZIZ13 | ICARDA | HUBARA-5/PASTOR-2 |
| *115* | 157:ZIZ13 | ICARDA | HUBARA-3/SHUHA-4//PASTOR-2 |
| *116* | 158:ZIZ13 | ICARDA | KATILA-17/DEEK-2/8/VEE'S'/7/CEBECO148/3/RON/CHA//BB/NOR67/5/HK/38MA/4/4777//REI/Y/3/KT/6/TUCAN'S' |
| *117* | 159:ZIZ13 | ICARDA | HUBARA-2/QAFZAH-21//DOVIN-2 |
| *118* | AJANA | AUSTRALIA | BLADE/2*KULIN |
| *119* | AMERY | AUSTRALIA | LR21-SRX/2*SHORTIM//3*BODALLIN |
| *120* | AROONA | AUSTRALIA | LERMA ROJO-64//NORIN-10/BREVOR-14/3/3*ANDES(WW15)/4/RAVEN |
| *121* | ARRINO | AUSTRALIA | 77W:660/ERADU |
| *122* | AXE | AUSTRALIA | (DH)RAC-875//EXCALIBUR/KUKRI/3/RAC-875//EXCALIBUR/KUKRI |
| *123* | BANKS | AUSTRALIA | PWTH/(SIB)CONDOR//2*CONDOR |
| *124* | BAXTER | AUSTRALIA | INIA-66/GAMUT//COOK/4/JUPATECO/3/LERMA-ROJO-64/SONORA-64-A//(SIB)TIMGALEN |
| *125* | BINNU | AUSTRALIA | ARRINO/(Y89-4034) ERADU*4/VPM1 |
| *126* | BODALLIN | AUSTRALIA | BOKAL/SIETE-CERROS-66 |
| *127* | BT SCHOMBURGK | AUSTRALIA | HALBERD/AROONA//3*SCHOMBURGK |
| *128* | BUMPER | AUSTRALIA | EXPRESS//PFAU/REEVES |
| *129* | CAMM | AUSTRALIA | VPM1.5*COOK/4*SPEAR |
| *130* | CARNAMAH | AUSTRALIA | BOLSENA-1CH/77W:660 |
| *131* | CASCADES | AUSTRALIA | AROONA*3//TADORNA/INIA66 |
| *132* | CHARA | AUSTRALIA | BD-225/CD-87 |
| *133* | COBRA | AUSTRALIA | (DER)WESTONIA |
| *134* | CRANBROOK | AUSTRALIA | WREN,MEX//CIANO-67(SIB)/NOROESTE-66/3/ZAMBEZ |
| *135* | CUNDERDIN | AUSTRALIA | CRANBROOK SISTER/SUNFIELD SISTER |
| *136* | DRYSDALE | AUSTRALIA | QUARRION/2*HARTOG |
| *137* | EAGLE ROCK | AUSTRALIA | SUNELG/2*BLADE |
| *138* | EGA BLANCO | AUSTRALIA | BOBWHITE(SIB)/NARIS-HUNTSMAN//CRANBROOK/VICAM-71(83-Z-1288)/3/(IW-1266)PFAU |
| *139* | EGA BONNIE ROCK | AUSTRALIA | SR9E.3*WARIGAL..3*AROONA (83Z:1048)/(82W:1097)3AG3.4*CONDOR..3*MILLEWA.3.BODALLIN |
| *140* | EGA CASTLE ROCK | AUSTRALIA | 3AG3.4*COOK/3*CASCADES |
| *141* | EGA 2248 | AUSTRALIA | 3AG3/3*HALBERD//4*TINCURRIN |
| *142* | ENVOY | AUSTRALIA | N/A |
| *143* | ERADU | AUSTRALIA | CIANO-67/GAMENYA |
| *144* | ESPADA | AUSTRALIA | (DH)RAC-875/KRICHAUFF//EXCALIBUR/KUKRI/3/RAC-875/KRICHAUFF/4/RAC-875//EXCALIBUR/KUKRI |
| *145* | ESTOC | AUSTRALIA | STYLET//(CO-6143)VM-931/RAC-935 |
| *146* | EXCALIBUR | AUSTRALIA | RAC-177(SR26)/UNICULM-492//RAC-311-S |
| *147* | FRAME | AUSTRALIA | MOLINEUX/3*DAGGER |
| *148* | GLADIUS | AUSTRALIA | (DH)RAC-875/KRICHAUFF//EXCALIBUR/KUKRI/3/RAC-875/KRICHAUFF/4/RAC-875//EXCALIBUR/KUKRI |
| *149* | HARPER | AUSTRALIA | YITPI/STYLET |
| *150* | HARTOG | AUSTRALIA | VICAM-711/CIANO"S"/SIETECERROS/3/KLYNSN/BLUEBIRD |
| *151* | KENNEDY | AUSTRALIA | VEERY#5/HARTOG |
| *152* | KING ROCK | AUSTRALIA | N/A |
| *153* | KUKRI | AUSTRALIA | MADDEN/6*RAC-177//GRAJO/76-ECN-44 |
| *154* | KULIN | AUSTRALIA | BODALLIN SIB//(HYDEN SIB)GAMENYA/INIA-66 |
| *155* | MACE | AUSTRALIA | WYALKATCHEM/STYLET//WYALKATCHEM |
| *156* | MAGENTA | AUSTRALIA | CARNAMAH/TAMMIN-18 |
| *157* | MILLEWA | AUSTRALIA | SONORA-64/YAQUI-50-ENANO//GABOTO/II-8156 |
| *158* | MITRE | AUSTRALIA | JANZ/BEULAH |
| *159* | PERENJORI | AUSTRALIA | BODALLIN/HYDEN |
| *160* | SAPPHIRE | AUSTRALIA | GBA-008/JANZ |
| *161* | SCOUT | AUSTRALIA | SUNSTATE/QH71-6//YITPI |
| *162* | SENTINAL | AUSTRALIA | N/A |
| *163* | SIVERSTAR | AUSTRALIA | PAVON"S"/(TM56)COCAMBA SIB |
| *164* | SPITFIRE | AUSTRALIA | DRYSDALE/KUKRI |
| *165* | STRZELECKI | AUSTRALIA | N/A |
| *166* | STYLET | AUSTRALIA | MOLINEUX/2*TRIDENT |
| *167* | SUNBROOK | AUSTRALIA | HARTOG*2/SUNECA |
| *168* | SUNGUARD | AUSTRALIA | SUN-289-E/SR2-JANZ |
| *169* | SUNTOP | AUSTRALIA | TESTED AS SUN595B AGT INBRED/CIMMYT INBRED |
| *170* | SUNVEX | AUSTRALIA | CNT-3/4*3765//2*CUNNINGHAM/3/2*SUNVALE |
| *171* | SUNZELL | AUSTRALIA | SUNBROOK*3/SUNSTATE |
| *172* | TAMMARIN ROCK | AUSTRALIA | SKOROSPELKA.4*LANCE:3*BODALLIN(81Y:970)/KALANNIE |
| *173* | TAMMIN | AUSTRALIA | BODALLIN//ERADU SIB/XBVT223/3/ATLAS66/2*MADDEN |
| *174* | TROJAN | AUSTRALIA | LPB 00LR000041/SENTINEL |
| *175* | VENTURA | AUSTRALIA | SUNVALE/ROWAN |
| *176* | WALLUP | AUSTRALIA | WYALKATCHEM/CHARA |
| *177* | WAWHT2046 | AUSTRALIA | AUS22857/KULIN/BLADE |
| *178* | WAWHT2074 | AUSTRALIA | N/A |
| *179* | WESTONIA | AUSTRALIA | SPICA/TIMGALEN(QT2085-20)/TOSCA(CO1190-203)//(84W127-501)CRANBROOK:JACUP*2/BOBWHITE |
| *180* | WYALKATCHEM | AUSTRALIA | MACHETE//(84W129-504)GUTHA/JACUP*2(11ISEPTON135)IASSUL/H567-71 |
| *181* | YANDANOOKA | AUSTRALIA | CALINGIRI/WAWHT-1137//38-W-386443 |
| *182* | YOUNG | AUSTRALIA | VPM-1/3*BEULAH//SILVERSTAR |
| *183* | ATTILA | MEXICO | NORD-DESPREZ/VG-9144//KALYANSONA/BLUEBIRD/3/YACO/4/VEERY-5 |
| *184* | BABAX | MEXICO | BOBWHITE/NACOZARI-76//VEERY/3/BLUEJAY/COCORAQUE-75 |
| *185* | BERKUT | MEXICO | IRENA/BAVIACORA-M-92//PASTOR |
| *186* | CHINO466 | CHINA | N/A |
| *187* | DUCULA | MEXICO | HUACAMAYO/TANORI-71-RESEL/3/ARTHUR*2/SIETE-CERROS-66//NACOZARI-76/4/ICTA-SARA-82 |
| *188* | INIA66 | MEXICO | LERMA-ROJO-64/SONORA-64 |
| *189* | KAUZ | MEXICO | JUPATECO-73/(SIB)BLUEJAY//URES-81 |
| *190* | PFAU | MEXICO | HORK(SIB)/YAMHILL//KALYANSONA/BLUEBIRD |
| *191* | SOKOLL | MEXICO | ASTOR/3/ALTAR-84/AE.SQ(TR.TA)//OPATA-M-85 |
| *192* | 6HRWSN 098 | CIMMYT | N/A |
| *193* | 6HRWSN 125 | CIMMYT | GOLDEN-VALLEY/AZTECA-67//MUSALA/3/DODO/4/BOBWHITE |
| *194* | 7HRWSN 108 | CIMMYT | N/A |
| *195* | 30 ZJN09 | CIMMYT | CS/TH.CU//GLEN/3/ALD/PVN/4/NINGMAI NO.4/OLESON//ALD/YANGMAI NO.4 |
| *196* | ZEE10 Qno 133 | CIMMYT | SUN371A*2/3/CHEN/AE.SQ//WEAVER |
| *197* | ZEE10 Qno 77 | CIMMYT | VEE/MJI//2*TUI/3/2*PASTOR/4/BERKUT/5/PFAU/MILAN |
| *198* | ZEE10 Qno 95 | CIMMYT | FRET2//SKAUZ*2/FCT/3/FILIN/2*PASTOR |
| *199* | ZJN11 Qno 104 | CIMMYT | CROC_1/AE.SQUARROSA (213)//PGO/3/CMH81.38/2*KAUZ/4/BERKUT |
| *200* | ZJN11 Qno 24 | CIMMYT | KS82142/2*WBLL1 |
| *201* | ZJN11 Qno 29 | CIMMYT | PASTOR//HXL7573/2*BAU/3/SOKOLL/WBLL1 |
| *202* | ZJN11 Qno 46 | CIMMYT | SOKOLL//SLVS/PASTOR/3/ATTILA*2//CHIL/BUC |
| *203* | ZJN11 Qno 47 | CIMMYT | SOKOLL//W15.92/WBLL1 |
| *204* | ZJN11 Qno 70 | CIMMYT | VEE/MJI//2*TUI/3/2*PASTOR/4/BERKUT |
| *205* | ZJN12 Qno 9 | CIMMYT | HUANIL//2*WBLL1*2/KUKUNA |
| *206* | ZVS09 Qno 125 | CIMMYT | DVERD_2/AE.SQUARROSA (214)//2*BCN/3/CALINGIRI |
| *207* | ZVS09 Qno 133 | CIMMYT | MILAN/DUCULA/4/CROC_1/AE.SQUARROSA (205)//KAUZ/3/SASIA |
| *208* | ZVS09 Qno 191 | CIMMYT | CALINGIRI/SOKOLL |
| *209* | ZVS09 Qno 33 | CIMMYT | SHARPSHOOTER/BRBT2 |
| *210* | ZWB11 Qno 124 | CIMMYT | TRCH/HUIRIVIS #1 |
| *211* | ZWB11 Qno 172 | CIMMYT | WAXWING/4/BL 1496/MILAN/3/CROC_1/AE.SQUARROSA (205)//KAUZ/5/FRNCLN |
| *212* | ZWB11 Qno 56 | CIMMYT | N/A |
| *213* | ZWB11 Qno 95 | CIMMYT | MUNAL #1/FRANCOLIN #1 |
| *214* | ZWC08 Qno 300 | CIMMYT | BETTY/3/CHEN/AE.SQ//2*OPATA |
| *215* | ZWE08 Qno 109 | CIMMYT | ATTILA*2/PBW65//BERKUT |
| *216* | ZWE10 Qno 30 | CIMMYT | SOKOLL//SUNCO/2*PASTOR |
| *217* | ZWW09 Qno 157 | CIMMYT | BERKUT/EXCALIBUR |
| *218* | ZWW09 Qno 177 | CIMMYT | SOKOLL/EXCALIBUR |
| *219* | ZWW09 Qno 59 | CIMMYT | KLDR/PEWIT1//MILAN/DUCULA |
| *220* | ZWW09 Qno 72 | CIMMYT | SOKOLL/EXCALIBUR |
| *221* | ZWW10 Qno 127 | CIMMYT | CUNNINGHAM/4/SNI/TRAP#1/3/KAUZ*2/TRAP//KAUZ |
| *222* | ZWW10 Qno 133 | CIMMYT | N/A |
| *223* | ZWW10 Qno 139 | CIMMYT | SOKOLL*2/4/CHEN/AEGILOPS SQUARROSA (TAUS)//FCT/3/STAR |
| *224* | ZWW10 Qno 155 | CIMMYT | PASTOR*2/BAV92/5/FRET2*2/4/SNI/TRAP#1/3/KAUZ*2/TRAP//KAUZ |
| *225* | ZWW10 Qno 157 | CIMMYT | TUKURU/4/CROC_1/AE.SQUARROSA (224)//YACO/3/MUNIA/5/BABAX/LR42//BABAX |
| *226* | ZWW10 Qno 29 | CIMMYT | NSM*4/14-2//FRTL/2*PIFED/3/VORB |
| *227* | ZWW10 Qno 31 | CIMMYT | BABAX/LR42//BABAX/3/BABAX/LR42//BABAX/4/T.DICOCCON PI94625/AE.SQUARROSA (372)//3*PASTOR/5/T.DICOCCON PI94625/AE.SQUARROSA (372)//3*PASTOR |
| *228* | ZWW10 Qno 5 | CIMMYT | POTCH93/4/MILAN/KAUZ//PRINIA/3/BAV92/5/MILAN/KAUZ//PRINIA/3/BAV92 |
| *229* | ZWW10 Qno 51 | CIMMYT | ACHTAR/4/MILAN/KAUZ//PRINIA/3/BAV92 |
| *230* | ZWW10 Qno 52 | CIMMYT | FDC36//ATTILA*2/PBW65 |
| *231* | ZWW10 Qno 60 | CIMMYT | MILAN/KAUZ//PRINIA/3/BAV92/4/ATTILA/BAV92//PASTOR/5/CNO79//PF70354/MUS/3/PASTOR/4/BAV92 |
| *232* | ZWW10 Qno 76 | CIMMYT | SOKOLL*2/ROLF07 |
| *233* | ZWW10 Qno 79 | CIMMYT | GK ARON/AG SECO 7846//2180/4/2*MILAN/KAUZ//PRINIA/3/BAV92 |
