# Supplementary material for: Phenotypic Evaluation and Genetic Analysis of Seedling Emergence in a Global Collection of Wheat Genotypes (Triticum aestivum L.) Under Limited Water Availability
Source: Front Plant Sci. 2021 Dec 24;12:796176. doi: 10.3389/fpls.2021.796176 (PMC8739788; doi:10.3389/fpls.2021.796176)
Supplement: Supplementary file 2 [file Table_2.DOCX]

| **Trait** | **QTL** | **Chromosome** | **SNP id** | **SNP name** | **SNP^a^** | **IGWSC-bp^b^** | ***R*^2^** | **MAF^c^** | **Allele effect estimate %^d^** | ***p*-value** | **-log_10_(*p*)** |
| --- | --- | --- | --- | --- | --- | --- | --- | --- | --- | --- | --- |
| Coleoptile length | *QCL.daw.1B-1* | 1B | *IWB26466* | Excalibur_c43567_850 | [**A**/G] | 7,995,497 | 0.06 | 0.39 | -28.30 | 1.98E-05 | 4.70 |
|  | *QCL.daw.1B-2* | 1B | *IWB74039* | Tdurum_contig98378_452 | [**A**/G] | 44,067,483 | 0.06 | 0.31 | -24.20 | 2.95E-05 | 4.53 |
|  | *QCL.daw.4B* | 4B * | *IWB70672* | Tdurum_contig41902_1524 | [T/**C**] | 24,556,539 | 0.06 | 0.06 | 32.87 | 1.55E-05 | 4.81 |
| Shoot length | *QSL.daw.1B-1* | 1B | *IWB74039* | Tdurum_contig98378_452 | [**A**/G] | 44,067,483 | 0.08 | 0.31 | -63.90 | 7.02E-05 | 4.15 |
|  | *QSL.daw.1B-2* | 1B * † | *IWB14703* | CAP8_c3752_330 | [T/**C**] | 667,966,067 | 0.08 | 0.12 | 69.20 | 7.27E-05 | 4.14 |
|  |  | 1B * † | *IWB20729* | Ex_c4436_1947 | [A/**G**] | 668,124,283 | 0.10 | 0.13 | 76.35 | 5.66E-06 | 5.25 |
|  |  | 1B * † | *IWA3892* | wsnp_Ex_c4436_7981037 | [A/**G**] | 668,124,497 | 0.10 | 0.16 | 73.97 | 1.16E-05 | 4.93 |
|  | *QSL.daw.4B* | 4B * | *IWB70672* | Tdurum_contig41902_1524 | [T/**C**] | 24,556,539 | 0.09 | 0.06 | 88.35 | 2.50E-05 | 4.60 |
|  | *QSL.daw.6A* | 6A * | *IWB71707* | Tdurum_contig46670_1680 | [T/**C**] | 599,049,790 | 0.09 | 0.38 | 44.65 | 6.01E-05 | 4.22 |
|  |  | 6A * | *IWB33787* | GENE-4118_142 | [T/**C**] | 599,050,251 | 0.09 | 0.41 | 45.86 | 2.94E-05 | 4.53 |
|  |  | 6A * | *IWB71705* | Tdurum_contig46670_1132 | [A/**G**] | 599,050,658 | 0.09 | 0.40 | 46.21 | 2.40E-05 | 4.62 |
| Coleoptile cross-section area | *QCSA.daw.1A* | 1A * | *IWA4852* | wsnp_Ex_c8885_14842394 | [T/**C**] | 352,173,604 | 0.08 | 0.43 | 0.43 | 2.12E-05 | 4.67 |

**Table S2.** SNP marker associations for coleoptile length, shoot length and coleoptile cross-section area for 233 wheat lines from different regions of the world, evaluated in a controlled environment.

^a^IWGSC: Desirable SNP for increased coleoptile length, shoot length and coleoptile cross-section area based on effect estimate is in bold and underlined.

^b^Base pair (bp) location of the single base change in the IWGSC RefSeq v1.0.

^c^MAF: minor allele frequency.

^d^The effect estimates the difference between the average phenotypic values of the homozygous A genotype relative to the homozygous B genotype.

*MTA in linkage disequilibrium with previously reported pre-harvest sprouting and dormancy QTL or genes (**Table S2**)

†MTA in LD with previously reported coleoptile length or shoot length QTL (**Table S2**)
