# Supplementary figures and images for: Phenotypic Evaluation and Genetic Analysis of Seedling Emergence in a Global Collection of Wheat Genotypes (Triticum aestivum L.) Under Limited Water Availability
Source: Front Plant Sci. 2021 Dec 24;12:796176. doi: 10.3389/fpls.2021.796176 (PMC8739788; doi:10.3389/fpls.2021.796176)

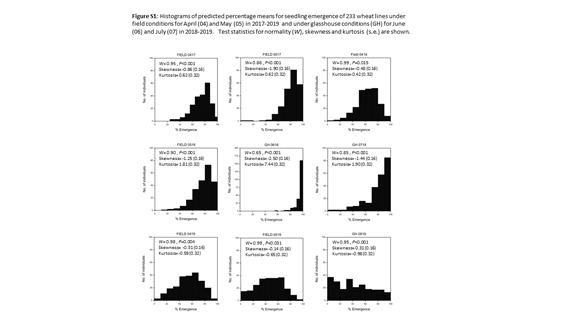

Supplement: Supplementary file 4 [file Image_1.TIF]
