# Supplementary material for: Phenotypic Evaluation and Genetic Analysis of Seedling Emergence in a Global Collection of Wheat Genotypes (Triticum aestivum L.) Under Limited Water Availability
Source: Front Plant Sci. 2021 Dec 24;12:796176. doi: 10.3389/fpls.2021.796176 (PMC8739788; doi:10.3389/fpls.2021.796176)
Supplement: Supplementary file 5 [file Presentation_1.PPTX]

## Slide 1
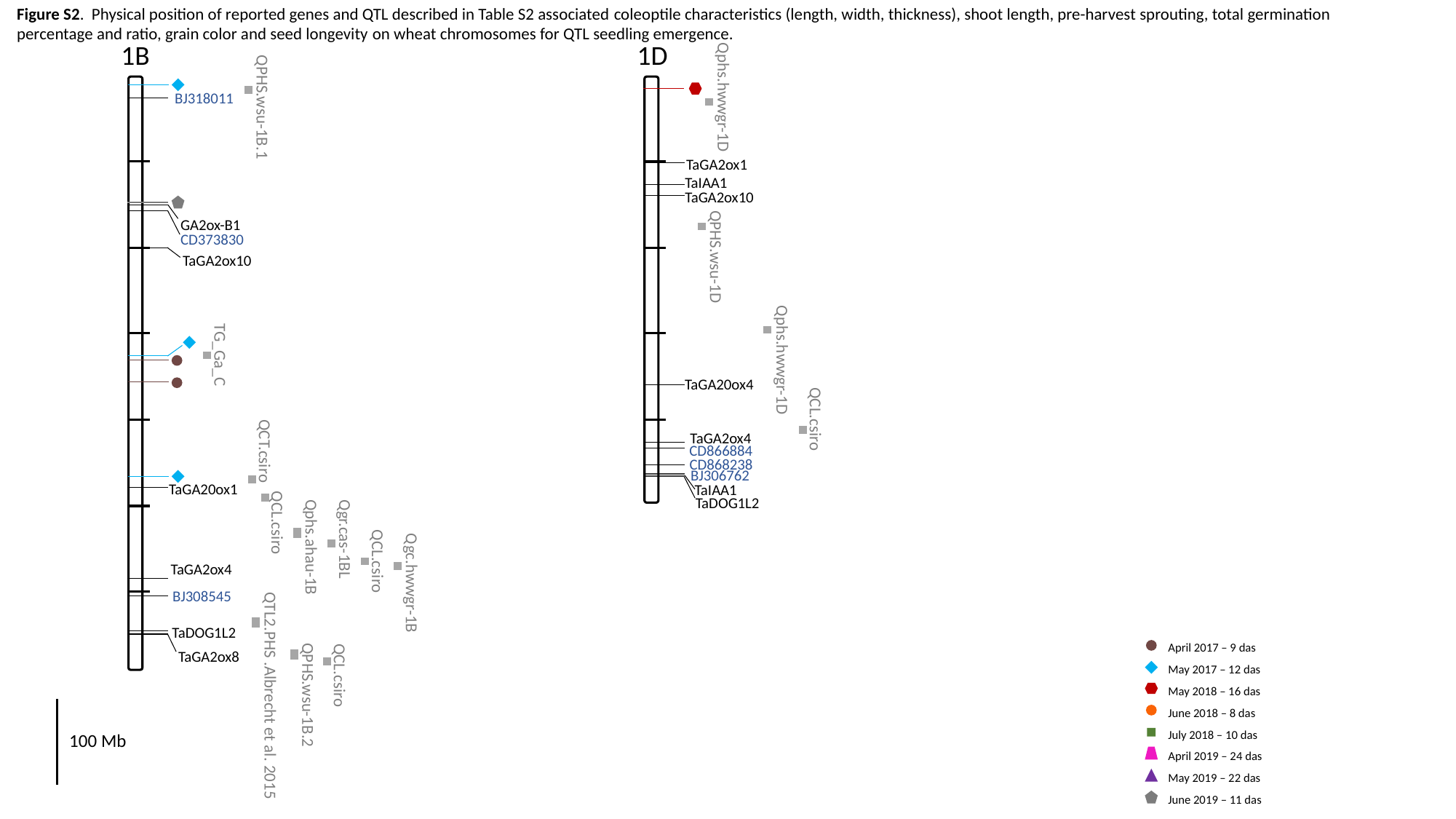

Figure S2. Physical position of reported genes and QTL described in Table S2 associated coleoptile characteristics (length, width, thickness), shoot length, pre-harvest sprouting, total germination percentage and ratio, grain color and seed longevity on wheat chromosomes for QTL seedling emergence.
Qphs.hwwgr-1D
1B
1D
QPHS.wsu-1B.1
BJ318011
TaGA2ox1
TaIAA1
TaGA2ox10
QPHS.wsu-1D
GA2ox-B1
CD373830
TaGA2ox10
Qphs.hwwgr-1D
TG_Ga_C
TaGA20ox4
QCL.csiro
QCT.csiro
TaGA2ox4
CD866884
CD868238
BJ306762
TaGA20ox1
TaIAA1
QCL.csiro
TaDOG1L2
Qgr.cas-1BL
Qphs.ahau-1B
QCL.csiro
Qgc.hwwgr-1B
TaGA2ox4
BJ308545
QTL2.PHS .Albrecht et al. 2015
TaDOG1L2
April 2017 – 9 das
May 2017 – 12 das
May 2018 – 16 das
June 2018 – 8 das
July 2018 – 10 das
April 2019 – 24 das
May 2019 – 22 das
June 2019 – 11 das
QPHS.wsu-1B.2
QCL.csiro
TaGA2ox8
100 Mb

## Slide 2
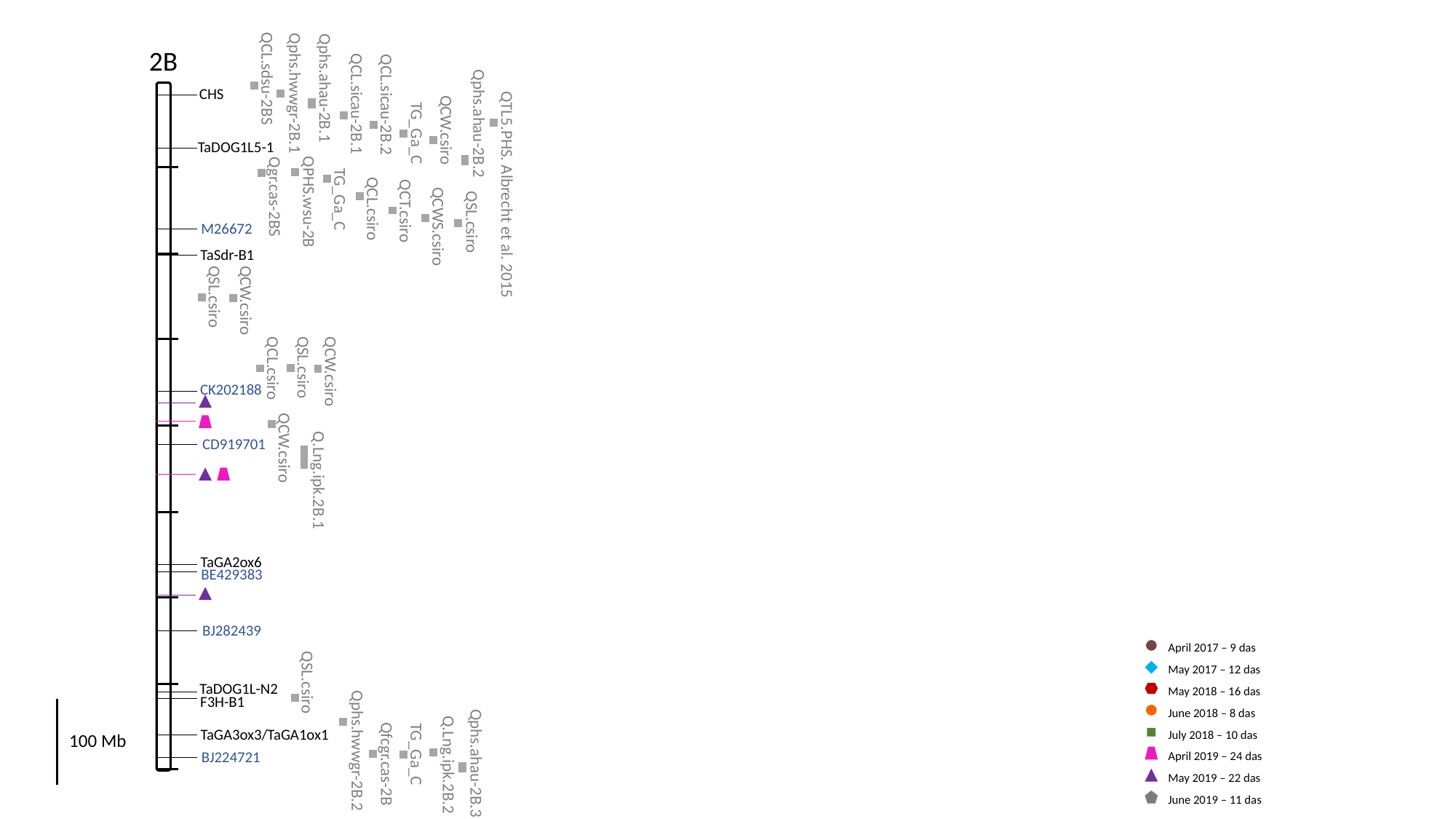

QCL.sdsu-2BS
Qphs.hwwgr-2B.1
Qphs.ahau-2B.1
2B
QCL.sicau-2B.1
QCL.sicau-2B.2
Qphs.ahau-2B.2
CHS
QTL5.PHS. Albrecht et al. 2015
QCW.csiro
TG_Ga_C
TaDOG1L5-1
QPHS.wsu-2B
Qgr.cas-2BS
TG_Ga_C
QCL.csiro
QCT.csiro
QCWS.csiro
QSL.csiro
M26672
TaSdr-B1
QCW.csiro
QSL.csiro
QSL.csiro
QCL.csiro
QCW.csiro
CK202188
QCW.csiro
Q.Lng.ipk.2B.1
CD919701
TaGA2ox6
BE429383
BJ282439
April 2017 – 9 das
May 2017 – 12 das
May 2018 – 16 das
June 2018 – 8 das
July 2018 – 10 das
April 2019 – 24 das
May 2019 – 22 das
June 2019 – 11 das
QSL.csiro
TaDOG1L-N2
Qphs.hwwgr-2B.2
F3H-B1
Qphs.ahau-2B.3
Q.Lng.ipk.2B.2
Qfcgr.cas-2B
TG_Ga_C
TaGA3ox3/TaGA1ox1
100 Mb
BJ224721

## Slide 3
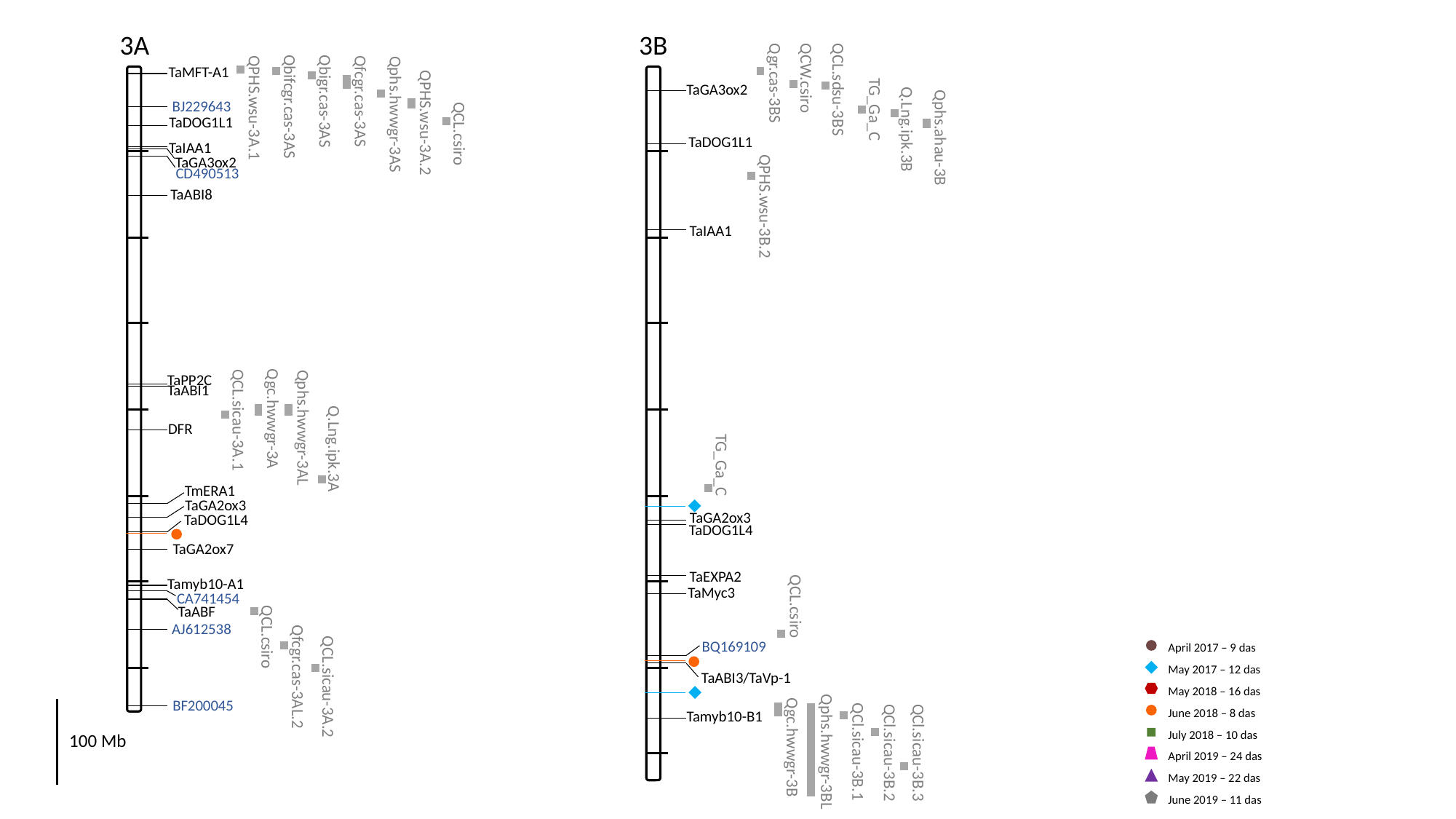

3A
3B
QCW.csiro
Qgr.cas-3BS
QCL.sdsu-3BS
Qbifcgr.cas-3AS
Qbigr.cas-3AS
QPHS.wsu-3A.1
Qfcgr.cas-3AS
Qphs.hwwgr-3AS
TaMFT-A1
QPHS.wsu-3A.2
TG_Ga_C
TaGA3ox2
Q.Lng.ipk.3B
Qphs.ahau-3B
BJ229643
QCL.csiro
TaDOG1L1
TaDOG1L1
TaIAA1
QPHS.wsu-3B.2
TaGA3ox2
CD490513
TaABI8
TaIAA1
Qgc.hwwgr-3A
QCL.sicau-3A.1
Qphs.hwwgr-3AL
TaPP2C
TaABI1
Q.Lng.ipk.3A
DFR
TG_Ga_C
TmERA1
TaGA2ox3
TaGA2ox3
TaDOG1L4
TaDOG1L4
TaGA2ox7
TaEXPA2
QCL.csiro
Tamyb10-A1
TaMyc3
CA741454
QCL.csiro
TaABF
AJ612538
Qfcgr.cas-3AL.2
QCL.sicau-3A.2
April 2017 – 9 das
May 2017 – 12 das
May 2018 – 16 das
June 2018 – 8 das
July 2018 – 10 das
April 2019 – 24 das
May 2019 – 22 das
June 2019 – 11 das
BQ169109
TaABI3/TaVp-1
Qphs.hwwgr-3BL
Qgc.hwwgr-3B
BF200045
QCl.sicau-3B.1
QCl.sicau-3B.2
QCl.sicau-3B.3
Tamyb10-B1
100 Mb

## Slide 4
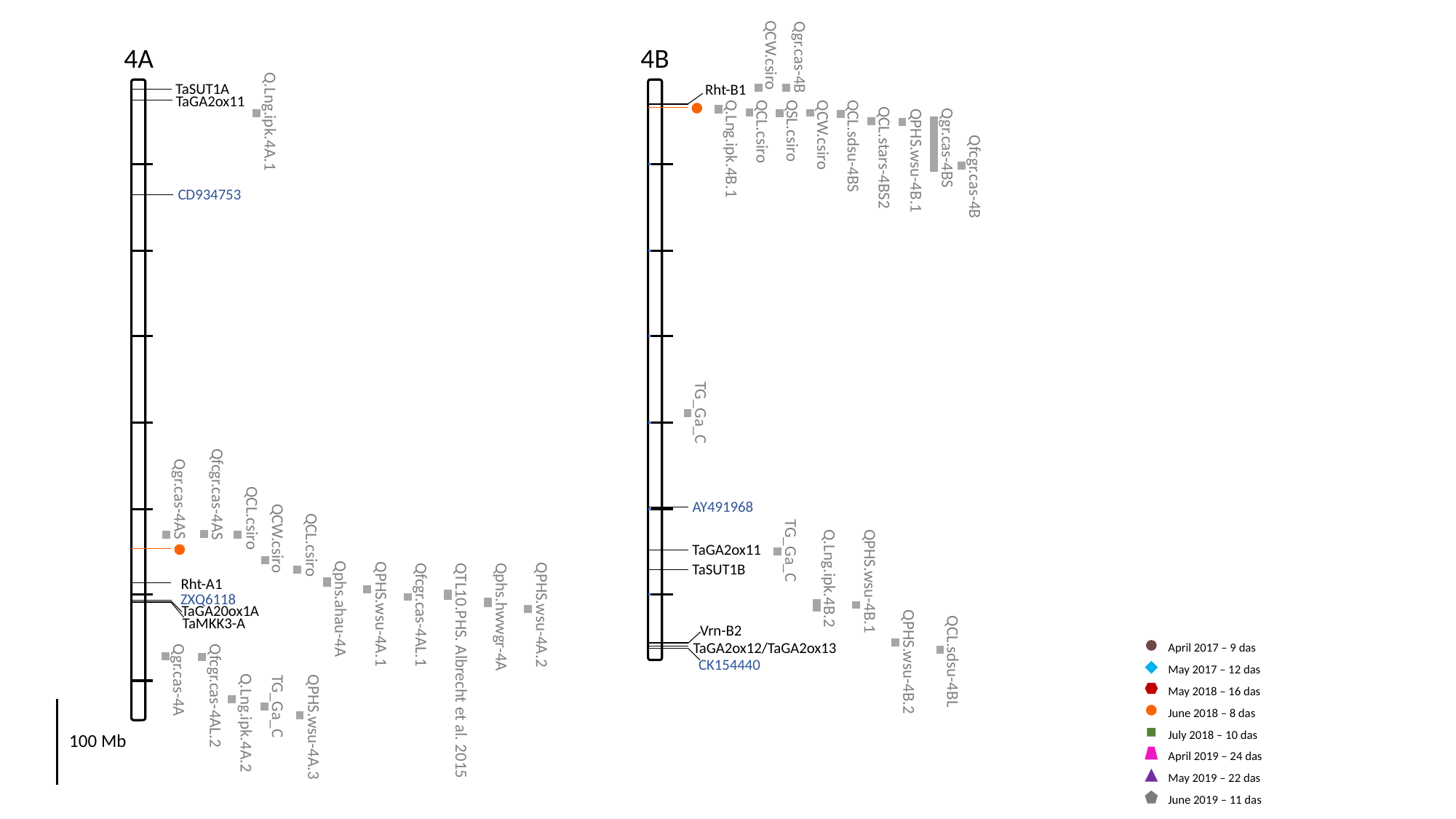

QCW.csiro
Qgr.cas-4B
4A
4B
Q.Lng.ipk.4A.1
TaSUT1A
Rht-B1
TaGA2ox11
QSL.csiro
Q.Lng.ipk.4B.1
QCL.sdsu-4BS
QCL.csiro
QCW.csiro
QCL.stars-4BS2
Qgr.cas-4BS
QPHS.wsu-4B.1
Qfcgr.cas-4B
CD934753
TG_Ga_C
Qfcgr.cas-4AS
Qgr.cas-4AS
QCL.csiro
AY491968
QCW.csiro
QCL.csiro
TG_Ga_C
QPHS.wsu-4B.1
Q.Lng.ipk.4B.2
TaGA2ox11
Qphs.ahau-4A
QPHS.wsu-4A.1
QPHS.wsu-4A.2
Qphs.hwwgr-4A
Qfcgr.cas-4AL.1
QTL10.PHS. Albrecht et al. 2015
TaSUT1B
Rht-A1
ZXQ6118
TaGA20ox1A
QPHS.wsu-4B.2
QCL.sdsu-4BL
TaMKK3-A
Vrn-B2
April 2017 – 9 das
May 2017 – 12 das
May 2018 – 16 das
June 2018 – 8 das
July 2018 – 10 das
April 2019 – 24 das
May 2019 – 22 das
June 2019 – 11 das
TaGA2ox12/TaGA2ox13
Qgr.cas-4A
Qfcgr.cas-4AL.2
CK154440
Q.Lng.ipk.4A.2
QPHS.wsu-4A.3
TG_Ga_C
100 Mb

## Slide 5
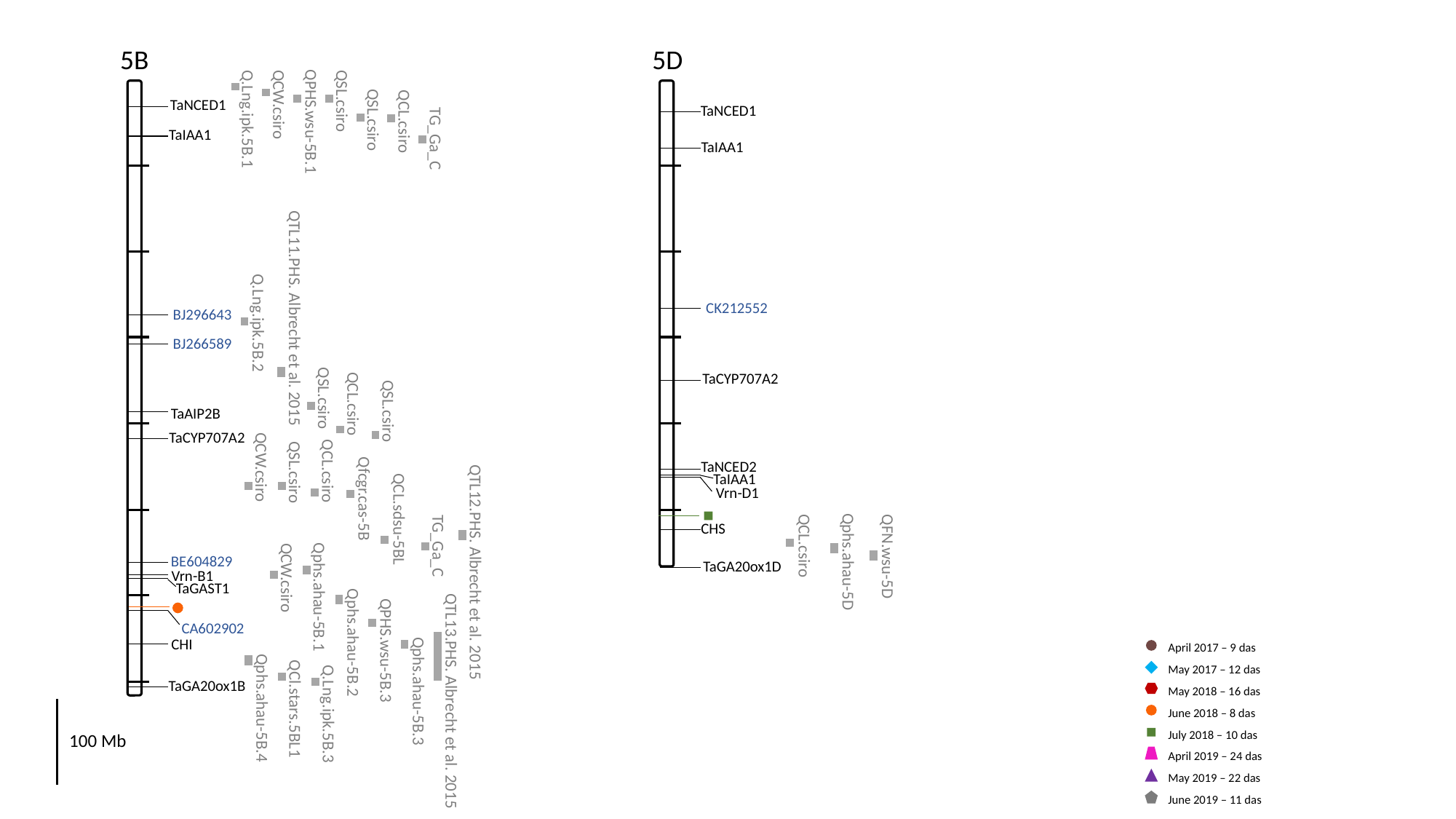

5B
5D
QPHS.wsu-5B.1
QCW.csiro
QSL.csiro
Q.Lng.ipk.5B.1
QSL.csiro
QCL.csiro
TaNCED1
TaNCED1
TG_Ga_C
TaIAA1
TaIAA1
QTL11.PHS. Albrecht et al. 2015
Q.Lng.ipk.5B.2
CK212552
BJ296643
BJ266589
QSL.csiro
QCL.csiro
TaCYP707A2
QSL.csiro
TaAIP2B
QCW.csiro
TaCYP707A2
QCL.csiro
QSL.csiro
Qfcgr.cas-5B
TaNCED2
QTL12.PHS. Albrecht et al. 2015
QCL.sdsu-5BL
TaIAA1
Vrn-D1
Qphs.ahau-5D
QCL.csiro
QFN.wsu-5D
TG_Ga_C
CHS
Qphs.ahau-5B.1
QCW.csiro
BE604829
TaGA20ox1D
Vrn-B1
TaGAST1
Qphs.ahau-5B.2
QTL13.PHS. Albrecht et al. 2015
QPHS.wsu-5B.3
CA602902
Qphs.ahau-5B.3
April 2017 – 9 das
May 2017 – 12 das
May 2018 – 16 das
June 2018 – 8 das
July 2018 – 10 das
April 2019 – 24 das
May 2019 – 22 das
June 2019 – 11 das
CHI
Qphs.ahau-5B.4
QCl.stars.5BL1
Q.Lng.ipk.5B.3
TaGA20ox1B
100 Mb

## Slide 6
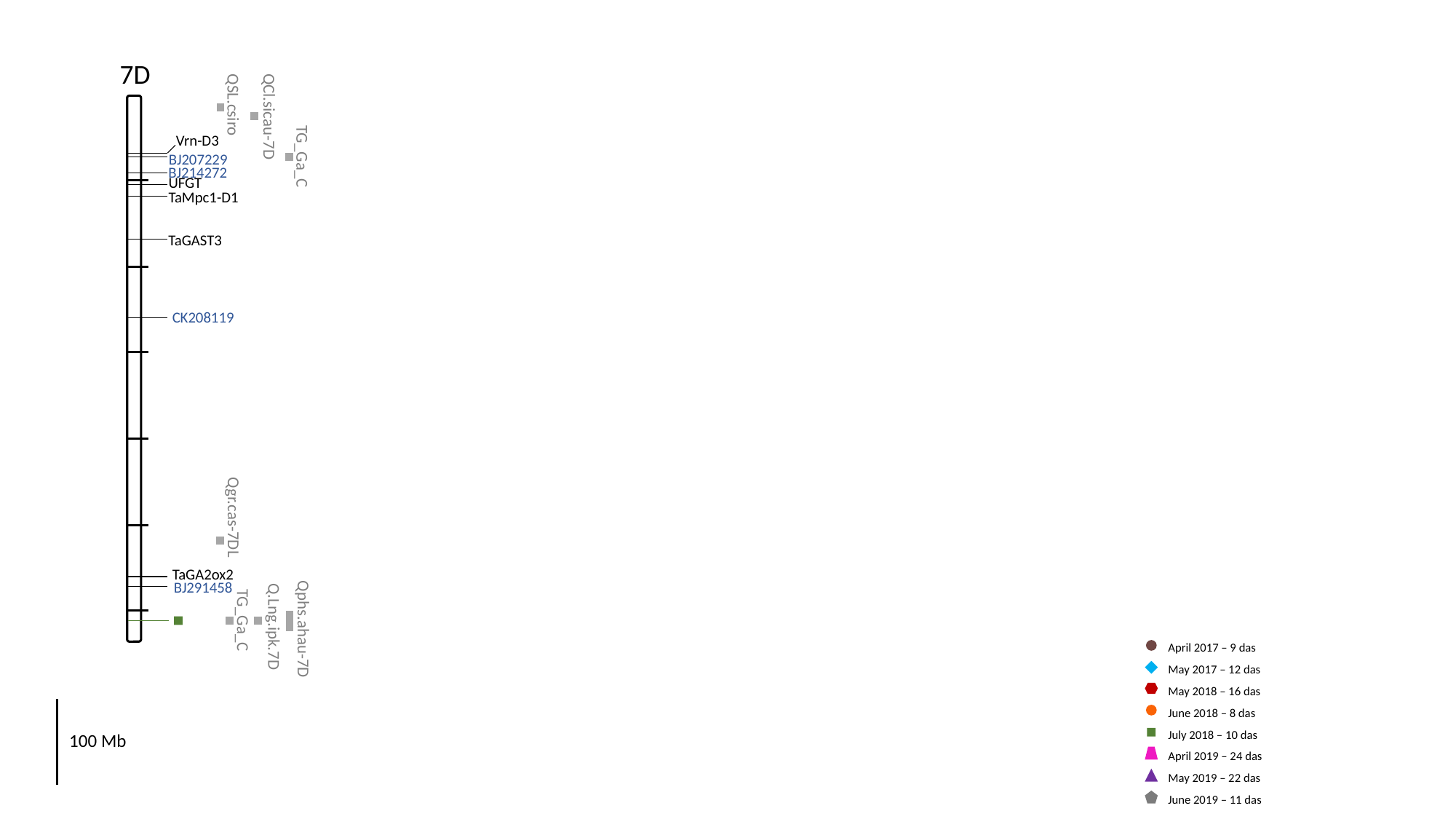

7D
QCl.sicau-7D
QSL.csiro
TG_Ga_C
Vrn-D3
BJ207229
BJ214272
UFGT
TaMpc1-D1
TaGAST3
CK208119
Qgr.cas-7DL
TaGA2ox2
Qphs.ahau-7D
BJ291458
Q.Lng.ipk.7D
TG_Ga_C
April 2017 – 9 das
May 2017 – 12 das
May 2018 – 16 das
June 2018 – 8 das
July 2018 – 10 das
April 2019 – 24 das
May 2019 – 22 das
June 2019 – 11 das
100 Mb
